# 3D printing-biomimetic local stiff niche enhances glycolysis to boost PDAC cell stem-like phenotype via N6-methyladenosine-suppressed YAP1 mRNA decay

**DOI:** 10.1016/j.mtbio.2025.102176

**Published:** 2025-08-05

**Authors:** Di Wu, Xiaoqi Guan, Tao Yang, Jiashuai Yan, Biwen Zhu, Junchao Zhou, Yibing Guo, Yuhua Lu

**Affiliations:** a Department of Hepatobiliary and Pancreatic Surgery, Affiliated Hospital of Nantong University, Medical School of Nantong University, Nantong 226001, PR China; b Research Center of Clinical Medicine, Affiliated Hospital of Nantong University, Nantong, Jiangsu province 226001, PR China

**Keywords:** PDAC stiffness heterogeneity, 3D printing, m6A, YAP1, Glycolysis, Stem-like phenotype

## Abstract

Cancer stem cells (CSCs), the primary source of therapy resistance in pancreatic ductal adenocarcinoma (PDAC), exist in a dynamic equilibrium through tumor microenvironment (TME)-driven plasticity. However, the stiffness heterogeneity of TME within PDAC functions on tumor cell stem-like phenotypes remains unclear. Bioinformatics, including Gene Ontology (GO) and Kyoto Encyclopedia of Genes and Genomes (KEGG) analysis, of CSCs identified from spatial transcriptomic and single-cell datasets of PDAC lesions exhibited activated mechanical and glycolytic pathways. Detected by the nano-indenter, PDAC tissue exhibited significant stiffness heterogeneity (200–6000 Pa). Further, biomimetic local stiffness niches were engineered using the digital light-processing (DLP) 3D-printing technology and the desmoplastic bioink (gelatin methacrylate & hyaluronic acid methacrylate, GelMA&HAMA) encapsulating PDAC cells, which permits modulation of mechanical properties without altering the biochemical ligand density. Stemness markers (NANOG, OCT4), glycolysis genes (HK2, LDHA), YAP1, and N6-methyladenosine (m6A) regulators (METTL14, IGF2BP3) were evaluated via qRT-PCR and immunofluorescence. Functional assays of glycolysis and stem-like phenotype were also conducted. Dot blot, RNA stability assay, western blot, and RIP assay were exploited to clarify the level and the function of m6A modification. The local stiff niche enhanced the stem-like phenotype of PDAC cells via YAP1-boosted glycolysis. Mechanistically, local stiff niche elevated the YAP1 level via m6A (METTL14/IGF2BP3)-stabilized YAP1 mRNA, linking mechanical inputs to glycolytic-stem-like phenotype adaptations. Collectively, the local stiff niches may drive the emergence of CSCs through epigenetic and metabolic reprogramming in PDAC mechanobiology. This provides new insights for developing more precise therapeutic strategies targeting PDAC mechanical heterogeneity.

## Highlights

• PDAC tissues exhibited remarkable stiffness heterogeneity with a range of 200–6000 Pa.
• The DLP 3D-printing permits tunable stiffness of the local niches without altering the desmoplastic bioink ligand density.
• The local stiff niche is an underestimated amplifier for the tumor stem-like phenotype via metabolic reprogramming in PDAC.
• m6A-YAP1 in stiff local niches reveals the plasticity of CSCs via mechano-epigenetic in the heterogeneous TME of PDAC.

## Graphical abstract

**Figure:**
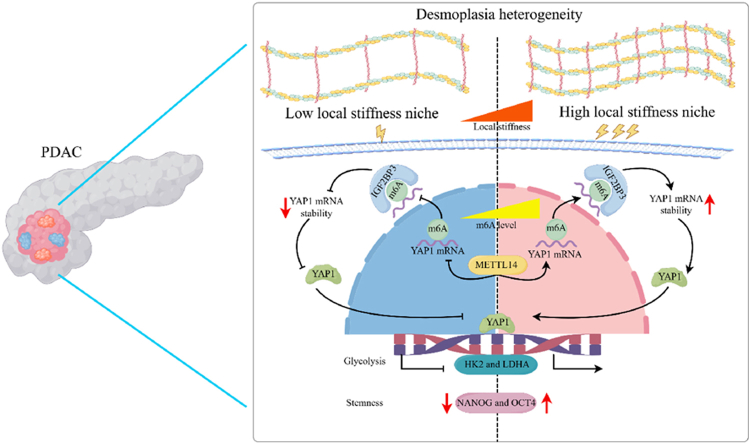

## 1 Introduction

Pancreatic ductal adenocarcinoma (PDAC) remains one of the most lethal malignancies with a dismal prognosis that is increasing on a global scale [1]. Typical therapy recalcitrance and relapse stem largely from the inherent intra-tumoral heterogeneity, where therapy-sensitive subpopulations are eradicated while resistant clones continue to do evil [2]. The cancer stem cells (CSCs), a critical insight into this heterogeneity, are characterized by self-renewal capabilities, which creates cell heterogeneity by establishing a differentiation hierarchy within tumors. These lead to the malignant cells that make up the majority of the tumor and produce a variety of distinct cell types [3]. Owing to the capacity to spread and initiate growth in remote locations, CSCs have been implicated in immune evasion and recurrence, which may represent the primary source of resistance to chemotherapy and radiation in PDAC [[4], [5], [6]]. Meanwhile, CSCs, gradually conceptualized as a functional state, exist in dynamic equilibrium with non-CSCs through microenvironment-driven plasticity [4,7]. Inflammatory and hyaluronic acid (HA)-rich tumor microenvironment (10.13039/501100010577TME) encourage the non-CSCs dedifferentiation into CSCs and support multipotent states through stromal crosstalk [8], underscoring the role of 10.13039/501100010577TME in shaping CSCs states.

The TME, as “soil” of cancer cells with high heterogeneity, drives malignant progression and therapeutic response by providing a multitude of altered biochemical and biomechanical cues [9]. Stiffness, a typically altered trait of solid malignancies that originates from the extracellular matrix (ECM), has drawn more attention as the function of the physical microenvironment within tumors was better understood. The PDAC TME represents a biomechanically active niche where desmoplasia-driven matrix stiffness ranks among the highest in solid tumors [10,11]. Although stiffness is believed to mediate the stem-like phenotype of PDAC cells, the associated investigations oversimplify the PDAC mechanical landscape by assuming a homogenous increase in stiffness [12], neglecting the primary tumors spatial heterogeneity of local niche stiffness, which may be caused by significant intra-tumoral heterogeneity, a hallmark of cancer [13]. It may be a critical knowledge gap impeding therapeutic development. There is reason to believe that the mechanical heterogeneity likely creates distinct local niches that differentially regulate tumor stem-like phenotype, and the corresponding mechano-transduction remains enigmatic in PDAC. Simultaneously, an advanced engineering model is necessary.

Signaling transduction is mostly dependent on the ECM, besides intercellular contact in TME [14], which influences cellular behavior through both biochemical and biomechanical cues [15]. Despite their importance, 2D culture, the traditional in vitro models, fail to mimic these intricate TME interactions accurately [16]. In the face of such hurdles, 3D bioprinting technology has emerged as a breakthrough and has been effectively harnessed for multiple cancer studies [[17], [18], [19]]. Digital light-processing (DLP) 3D printing technology is a light-based technique that can produce scaffolds with micron-scale resolution in just a few seconds. The continuous 3D printing process photopolymerizes an entire layer of biomaterial at the focal plane, allowing for the creation of tissue-engineered scaffolds with complex microarchitectures [20,21]. In the same time, the hydrogel-based bioinks also exerted on improving the simulation of TME characteristics. Our lab-developed dual-crosslinked gelatin methacrylate (GelMA) & hyaluronic acid methacrylate (HAMA) hydrogel system uniquely mimics PDAC ECM with excessive deposition of collagen I (COL I) and hyaluronic acid that concurrently results in the desmoplasia and the abnormal stiff tumor mass [[22], [23], [24]]. Combining DLP 3D printing, the system permits independent modulation of stiffness through ultraviolet light (UV) cross-linking duration at a constant concentration to eliminate confounding biochemical variables when interrogating stiffness-specific effects. These enable the construction of biomimetic local stiffness niches, simulating the biomechanical heterogeneity of PDAC TME more precisely.

The growth and resistance to treatment of CSCs are maintained by the abnormal metabolic states [4]. And the unique desmoplasia TME of PDAC strongly affects diffusion of oxygen and nutrients, which is accompanied by metabolic reprogramming, specifically a shift towards glycolysis (Warburg effect) [25,26]. Stem-like phenotypes in various types of tumor cells were boosted by glycolysis, including PDAC [23]. The crosstalk between metabolism and mechano-transduction has called more attention owing to the mechanical cues conveyed by signaling pathways that also mediate metabolic processes. Signals of mechano-transduction from TME stiffness in hepatocellular carcinoma trigger activation of MAPK/YAP pathway and accelerated aerobic glycolysis [27]. These further suggest that the local niches with specific stiffness are accompanied by particular metabolic pathways. Hence, we hypothesize that stiffness heterogeneity may drive metabolic-stem-like phenotype crosstalk via mechanosensitive pathways in PDAC.

The yes-associated protein 1 (YAP1) emerges as a prime candidate linking mechanical cues to CSCs phenotypes. This Hippo pathway effector transduces matrix stiffness signals, maintains stem-like properties in multiple cancers, and regulates glycolytic flux. It is essential for both in vitro organ-specific stem cell growth as organoids and in vivo tissue repair [28]. According to the research in hepatocellular carcinoma, matrix stiffness boosts the stem-like phenotype of tumor cells by YAP1 [29]. It also functioned as a key regulator for glycolysis in non-small cell lung cancer and colorectal cancer [30,31]. And YAP1 accounts for the aggressive behavior of PDAC cells [32]. Hence, it is reasonable to suppose that YAP1 is the hinge to link the glycolysis and stem-like phenotype within the PDAC heterogeneous stiffness TME. Meanwhile, epigenetic mechanisms in various cancers regulate YAP1. It is reported that METTL3 enhances the proliferation and migration of gastric cancer cells by modifying YAP1 through N6-methyladenosine (m6A) [33]. Furthermore, m6A-modified circTEAD1 stabilizes YAP1 mRNA, which contributes to the tumorigenesis in chordoma [34].

m6A is the predominant form of modification in mammalian mRNA. The abundance and effects of m6A on RNA are determined by the dynamic interplay between its methyltransferases (writers, e.g., METTL3, METTL14), binding proteins (readers, e.g., FTO, ALKBH5), and demethylases (erasers, e.g., IGF2BP1/2/3). “Readers” recognize the m6A mark induced by “writers” and “erasers” and exert control over mRNA transcription, splicing, subcellular distribution, stability, and translation. Accumulating evidence has suggested that altered m6A modifications are widely involved in the initiation and progression of cancer. It is important to note that m6A responds dynamically to stimuli from the microenvironment [35], which hints at the interaction between m6A and mechanobiology. Determining whether m6A modifications respond to the heterogeneous stiffness in the PDAC TME and subsequently regulate YAP1 levels is a critical knowledge gap.

In our research, we found that CSCs within PDAC primary and liver metastasis lesions displayed the activated biological processes of response to mechanical stimulus and glycolysis according to spatial transcriptomics and single-cell transcriptomics. To investigate whether and how the stiffness heterogeneity within PDAC exerts influence on tumor cell stem-like type, biomimetic local niches with low, medium, and high local stiffness were engineered based on the DLP 3D-printing technology and the desmoplastic bioink, which simulate mechanical heterogeneity according to the spatial stiffness variations mapped by the nano-indenter in human primary PDAC specimens. Mechanical priming in stiff local niches endows the acquisition of a stem-like phenotype by YAP1-induced glycolysis augmentation. Mechanistically, local stiff niche elevated the YAP1 level via m6A (METTL14/IGF2BP3)-suppressed YAP1 mRNA decay, linking mechanical inputs to glycolytic-stem-like phenotype adaptations, and offering evidence of epi-transcriptomic regulation in PDAC mechanobiology. Collectively, the local mechanically heterogeneous niches may drive the emergence of CSCs subpopulations via both epigenetic and metabolic reprogramming, offering novel perspectives for more precise therapeutic strategy development in PDAC.

## 2 Methods and materials

### 2.1 Materials and regents

Sodium hyaluronate (H293507), Methacrylic anhydrides (M102519), and Sodium hydroxide (NaOH, S111507) were purchased from Aladdin Co., Ltd. (Shanghai, China). Gelatin (from porcine, 73865) and Lithium phenyl-2, 4, 6-trimethylbenzoylphosphinate (LAP, 900889) were purchased from Sigma Aldrich (Shanghai, China). Gemcitabine (GEM, IG1120) were purchased from Solarbio (Beijing, China). 2-Deoxy-D-glucose (2-DG, HY-13966), Oligomycin (Oligo, HY-N6782), Verteporfin (VP, HY-B0146), PY60 (HY-141644), and Actinomycin D (Act D, HY-17559) were purchased from MedChemExpress (MCE, China).

### 2.2 Single-cell transcriptomics and spatial transcriptomics

The spatial transcriptomics data (GSE205354) of primary pancreatic ductal adenocarcinoma (PDAC) was obtained from published sources [36]. The statistics and visualization were performed by STOmicsDB platform [37]. The single-cell transcriptomics data of PDAC liver metastasis (GSE197177) derived from Shu Zhang et al. [38] and analyzed by Cell-omics Data Coordinate Platform (https://db.cngb.org/cdcp/) [39].

### 2.3 PDAC samples

The tumor tissues (n = 6) were acquired from PDAC patients with informed consent. The experimental protocol was conducted following relevant laws and approved by the ethics committee of the Affiliated Hospital of Nantong University (No. 2021-K136). All cases diagnosed by a qualified pathologist belong to the early stage according to the AJCC 8th tumor stage.

### 2.4 Masson’ s trichrome staining

After deparaffinizing paraffin sections in solution A overnight, the sections were stained with a B and C mixture solution (1:1) before rinsing and then submerged in solutions D and E for 6 min and 1 min, respectively. Following a 20 s staining in F solution, the sections were dehydrated in absolute ethanol, rinsed in 1 % acetic acid, and clarified in xylene for 5 min. Using a fluorescence microscope (Leica, Thunder, German), pictures were captured. ImageJ software was used to calculate the area and percentage of collagen.

### 2.5 Alcian blue staining

After deparaffinization, the paraffin sections were stained with Alcian blue (Servicebio, G1027, China) for 15 min, followed by Nuclear Fast Red (Servicebio, G1035, China) for 3 min. The sections were sealed with neutral resin following dehydration. Images were taken with fluorescence microscope (Leica, Thunder, German). The area and percentage of glycosaminoglycans (GAGs) were determined using ImageJ software.

### 2.6 Stiffness testing

The PDAC tissues were immersed in 0.9 % sodium chloride at 4 °C and tested within 2 h after harvest. The hydrogel samples were transferred to complete culture medium to achieve the swelling equilibrium before mechanical characterization. The stiffness of the samples was measured by the nano-indenter (PIUMA, Optics11, The Netherlands) with a 48 μm spherical tip (0.44 N/m spring constant). Indentation parameters: 20 μm displacement, 10 μm/s speed, 2 s relaxation. The samples were immersed in buffer solution at room temperature, ensuring that the nano-indenter tip was consistently maintained at a depth well below the buffer's surface throughout the procedure. Based on the force-displacement curves, Young’ s modulus was automatically determined by the PIUMA software (Optics11, v1).

### 2.7 Cell culture

Human PDAC cell lines, MIA-PACA2 and CFPAC-1 cells, were generously donated by the Chinese Academy of Sciences’ Stem Cell Bank. Cells were cultivated in accordance with the guidelines. Briefly, MIA-PACA2 cells cultured with DMEM (Gibco, 12430054, USA) contained 2.5 % (v/v) heat-inactivated horse serum (Gibco, 26050070, USA) and 1 % (v/v) sodium pyruvate (Gibco, 11360070). And CFPAC-1 cells with IMDM (Corning, 10-016-CV, USA). Both cell lines were cultured at 37 °C and 5 % CO_2_ atmosphere with supplementary 10 % (v/v) prime fetal bovine serum (Vazyme, F103-01, China). Trypsin-0.25 % EDTA (KeyGEN BioTECH, KGL2102-100, China) regularly detaches cells when they reach the logarithmic phase.

### 2.8 Desmoplastic bio-ink preparation

Gelatin Methacrylate (GelMA) and hyaluronic Acid Methacrylate (HAMA) were synthesized according to our previous study [22]. The LAP was dissolved with PBS in the dark to obtain a final concentration of 0.5 %. To achieve a final concentration of 0.5 %, the LAP was dissolved in PBS while protected from light. 7.5 % (w/v) GelMA and 1 % (w/v) HAMA were dissolved in LAP and stirred in a 37 °C water bath, and the solution was further encapsulated the PDAC cell lines at 5 × 10^5^ cells/mL to prepare the desmoplastic bioink.

### 2.9 Scanning electron microscope (SEM)

After being fast frozen and freeze-dried, the samples were trimmed to reveal the pores on their inner surface and then coated with a gold film. The micro-morphology was examined using scanning electron microscopy (Zeiss, Gemini 300, Germany). Porosity was assessed via Image J.

### 2.10 Rheological behavior and viscosity

The in-situ photo-rheology of hydrogel encapsulated cells was conducted using a rotational rheometer (Thermo Scientific, Haake RS6000, USA) at a frequency of 5 rad/s and a strain of 1 %. The measurements were performed with a 20 mm parallel plate at 37 °C. Ultra-low viscosity silicone oil was applied to the edges of the sample to keep it from drying out. At a frequency of 1 Hz, the storage modulus (G′) and loss modulus (G″) were measured. The rotational viscosity-shear measurements were also performed with shear rates from 0.1 to 100 s^−1^ under 37 °C.

### 2.11 3D printing

The appearance of the 3D-printed niches was designed as a cylinder (diameter = 8 mm, height = 4 mm) with four internal holes (diameter = 1.6 mm). A digital light-processing (DLP) 3D printer (EFL-BP8601 pro, EFL, China) was used for bioprinting. Before printing, the desmoplastic bio-ink as described above was mixed with a light absorber (at a final 0.05 % solution) and loaded into the ink tank. During printing, the model was printed at a UV light (405 nm) intensity of 20 mW/cm^2^ and irradiated for 6, 10, and 20 s. In total, 40 layers, each 100 μm-thick, were printed. The 3D-printed niches were gently removed to the 24-well plate, and complete culture medium was replaced three times within 3 h to remove redundant LAP and soluble polymer chains.

### 2.12 Swelling test

The printed models were continued to be cultured as above cell culture conditions until the it reached swelling equilibrium, then taken out and weighed at an appointed time. Ws and Wd were defined as the weights of swollen hydrogel and dry hydrogel, respectively. The swelling ratio was calculated according to the equation: Swelling ratio (%) = (Ws - Wd)/Wd × 100 %.

### 2.13 Degradation test

The printed models were continued to be cultured as above cell culture conditions. The fresh medium was replaced every 2 days. At the appointed time, the models were dried and weighed after the medium was removed. The degradability was calculated according to the equation: Weight ratio (%) = Wdd/Wdi × 100 %. Wdd and Wdi were defined as the dry weight after degradation and the weights of initial dry weight, respectively.

### 2.14 Biocompatibility

The biocompatibility of the PDAC cell lines within 3D printing biomimetic local stiffness niches was evaluated by the Calcein-AM/PI Double Staining Kit (Solarbio, IC4630, China) according to instructions. Briefly, the cells were incubated for 15 min at 37 °C without light in the Calcein AM and PI mixed solution. The living cells (green) and the dead cells (red) were imaged with the confocal microscope (Zeiss, LSM 900, Germany). Cell viability was quantified using Image J software.

### 2.15 Cell morphology

The cells were permeabilized with 0.1 % Triton X-100 for 20 min after being fixed for 15 min in 4 % paraformaldehyde. Followed by three PBST rinses and 1 h block in 5 % BSA. Before being counter-stained with DAPI, the cells were incubated with phalloidin (1:100, Solarbio, CA1610, China) at 37 °C for 30 min. Using a confocal microscope (Zeiss, LSM 900, Germany), pictures were taken.

### 2.16 Hematoxylin-eosin (H&E)

Paraffin sections were deparaffinized and then stained with the Hematoxylin-Eosin Stain Kit (Solarbio, G1120, China) following the guidelines of the manufacturer. Briefly, paraffin sections were deparaffinized and stained with hematoxylin solution for 15 min, followed by differentiation solution for 3 min. Re-dyeing with eosin Y aqueous solution for 1 min before dehydrating, trans-parenting by xylene, and sealing with neutral resin. Images were photographed using a microscope (Leica, Thunder, Germany).

### 2.17 RNA extraction and qRT-PCR

Total RNA was extracted via TRIZOL (Invitrogen, 343903, USA) and quantified with a spectrophotometer (NanoDrop One, Thermo, USA). Complementary DNA (cDNA) was amplified with a T100 Thermal Cycler (Bio-Rad, USA) according to the RevertAid First Strand cDNA Synthesis Kit (Thermo Scientific, K1622, USA). The primers were synthesized by Sangon Biotech (Shanghai, China), including NANOG: 5′-ACTGGCTGAATCCTTCCTCTCC-3′ and 5′-GCTGATTAGGCTCCAACCATACTC-3′; OCT4: 5′-GCAGAAGTGGGTGGAGGAAGC-3′ and 5′-GGTTGCCTCTCACTCGGTTCTC-3′; HK2: 5′-GGTTGCTTCTGGCTCCTCCTTC-3′ and 5′-TGCTGTGGTCTCTTCAGTCTATTGG-3′; LDHA: 5′-CAGCCCGATTCCGTTACCTAATGG-3′ and 5′-ACACCAGCAACATTCATTCCACTCC-3′; YAP1: 5′-ACAGCAGAACCGTTTCCCAGAC-3′ and 5′-TCATTCCATCTCCTTCCAGTGTTCC-3′; METTL14: 5′-TGGCAGTCAGAATGTAATACGGTCATC-3′ and 5′-GGTTGCCATGCCACAATCCTG -3′; IGF2BP3: 5′-GAGGCGCTTTCAGGTAAAATAG-3′ and 5′-AATGAGGCATATTTCGTAT-3′; β-actin: 5′-CCTGGCACCCAGCACAAT-3′ and 5′- GGGCCGGACTCGTCATAC-3′. The mRNA expression was carried out in triplicate by Blue SYBR Green qPCR Master Mix (Servicebio, G1006, China) and Studio Q5 (ABI, A28134, USA). The expression level was determined using the 2^−ΔΔCt^ method.

### 2.18 Immunofluorescence (IF)

All samples were fixed with 4 % paraformaldehyde, permeabilized with 0.2 % Triton X-100, and blocked with 10 % BSA at room temperature. Incubated with the primary antibodies overnight at 4 °C: NANOG (1:200, Abcam, ab109250, UK), OCT4 (1:250, Abcam, ab181557, UK), HK2 (1:200, Proteintech, 22029-1-AP, China), LDHA (1:300, Proteintech, 19987-1-AP, China), YAP1 (1:100, ABclonal, A1002, China), m6A (1:2000, Proteintech, 68055-1, China), METTL14 (1:500, Selleck, F1391, USA) and IGF2BP3 (1:100, Proteintech, 14642-1-AP, China). Rinsed with PBST 3 times, then incubated with multi-rAb™ CoraLite® Plus 647-Goat Anti-Rabbit Recombinant Secondary Antibody (H + L) (1:600, Proteintech, RGAR005, China) or multi-rAb™ CoraLite® Plus 647-Goat Anti-Mouse Recombinant Secondary Antibody (H + L) (1:600, Proteintech, RGAM005, China) for 2 h in the dark, counterstained with DAPI. Images were photographed via the confocal microscope (Zeiss, LSM 900, Germany).

### 2.19 Sphere forming assay

Cells were seeded in ultra-low attachment 96-well (NEST, 70111B01, China) at a density of 1000 cells per well in serum-free DMEM/F12 media. After 5 days, images were photographed using a fluorescence microscope (Leica, Thunder, Germany). Spheres larger than 50 μm in diameter were counted and the sphere formation efficiency was calculated as the number of spheres per well divided by the number of cells seeded per well.

### 2.20 Half maximal inhibitory concentration (IC_50_)

Cells were treated with gemcitabine (0.01 , 1 , 10 , 100 , and 1000 μM) for 48 h. CCK-8 (DOJINDO, CK04, Japan) was added and incubated according to the instructions of the manufacturer. The absorbance at 450 nm was measured using a microplate reader (BMG, CLARIOstarPlus, Germany), and the half maximal inhibitory concentration (IC_50_) was calculated by logarithmic regression using GraphPad Prism software (9.3).

### 2.21 Immunohistochemistry (IHC)

The specimens were fixed in formalin, embedded in paraffin, cut into 5 μm sections, and affixed to glass slides. The sections underwent a 10 min incubation in a peroxidase blocking agent, followed by PBS washing, a 60 min blockade with BSA, and an overnight incubation at 4 °C with primary antibodies: NANOG (1:400, Servicebio, GB11331, China), OCT4 (1:100, Proteintech, 11263-1-AP, China), HK2 (1:100, Servicebio, GB151063, China), LDHA (1:400, Servicebio, GB11342, China), YAP1(1:800, Servicebio, GB113975, China). Subsequently, the sections were incubated with an HRP-conjugated secondary antibody (1:200, Servicebio, GB23303 and GB23301, China) for 10 min at room temperature, underwent development with a diaminobenzidine solution, and were counterstained with hematoxylin. Imaging was photographed by a microscope (Leica, Thunder, German).

### 2.22 Animal experiments

All animal experiments were performed following the guidelines for the Care and Use of Laboratory Animals of Nantong University and were approved by the Animal Ethics Committee of Nantong University (No. S20241201-009). 3D-printed niches were subcutaneously transplanted into 5-week BALB/c nude mice. Tissues were harvested at day 14 for IHC analysis.

### 2.23 Glucose consumption

The glucose concentrations of the supernatant were measured via the Glucose Assay Kit with O-toluidine (Beyotime, S0201M, China) following the instructions of the manufacturer. Briefly, the supernatant of cells within 24 h was collected and mixed with the Glucose Assay Reagent. Then the mixture was reacted at 95 °C for 8 min, followed by cooling to 4 °C using a Thermal Cycler (Bio-Rad, T100, USA). Then the absorbance of the mixture at 630 nm was measured by a microplate reader (BMG, CLARIOstar^Plus^, Germany). The glucose concentration was determined by referencing a standard calibration curve and normalized by the protein concentration of the samples. The relative glucose consumption was normalized to the control group.

### 2.24 ATP level

The ATP level was evaluated by an ATP Assay Kit (Beyotime, S0026B, China) in accordance with the recommendation of the manufacturer. Briefly, add ATP detection reagent to the sample, and the chemiluminescence intensity was detected by a microplate reader (BMG, CLARIOstar^Plus^, Germany). ATP concentration was calculated according to the standard curve and normalized by the protein concentration of the samples. The relative ATP level for each sample was normalized to the control group.

### 2.25 Lactate production

The level of lactate was measured using a Lactic Acid (LA) Content Assay Kit (Solarbio, BC2235, China) according to the manufacturer's instructions. Firstly, samples were incubated with Reagent 1, Reagent 2, and Reagent 4 at 37 °C for 20 min. Then add Reagent 5 and Reagent 3 and incubate at 37 °C for 20 min in the dark. The pellet was collected by centrifuging at 10,000 rpm for 10 min and dissolved in ethanol. Then the absorbance at 570 nm was measured using a microplate reader (BMG, CLARIOstar^Plus^, Germany). The lactate concentration in the samples was calculated based on a standard curve and normalized by the protein concentration of the samples. The relative lactate production was normalized to the control group.

### **2.26** **TCGA database**s **and GEPIA tools**

RNA-sequencing expression profiles and corresponding clinical information for PDAC were downloaded from the TCGA dataset (https://portal.gdc.cancer.gov). The current-release GTEx datasets were obtained from the GTEx data portal website (https://www.gtexportal.org/home/datasets). The visualization of overall survival (OS) analysis, disease specific survival (DSS) analysis, and progress free interval (PFI) analysis was performed with R packages (survival [3.3.1], survminer [0.4.9], ggplot2 [3.3.6]). For Kaplan-Meier curves, p-values and hazard ratio (HR) with 95 % confidence interval (CI) were generated by log-rank tests and univariate Cox proportional hazards regression. The Gene Ontology (GO) and Kyoto Encyclopedia of Genes and Genomes (KEGG) enrichment were performed with R packages (cluster Profiler [4.4.4]). The genotype-tissue expression analysis and co-expression analysis were performed by GEPIA2 website tool (http://gepia.cancer-pku.cn/). p-value <0.05 was considered statistically significant.

### 2.27 SRAMP

The SRAMP database (Sequence-based RNA Adenosine Methylation Site Predictor) [40] was utilized to predict the m6A sites of YAP1. The prediction was performed using the full transcript mode of SRAMP (http://www.cuilab.cn/sramp). The input sequence of YAP1 was obtained from the NCBI database. The prediction parameters were set to default values, and the output included the predicted methylation sites and their corresponding scores.

### 2.28 m6A dot blot assay

m6A dot blot assay was performed according to the Rui Su et al. [41]. Briefly, mRNA samples were denatured at 65 °C for 10 min with RNA incubation buffer. An equal volume of chilled 20 × SSC buffer was then added before samples were spotted on the Amersham Hybond-N+ membrane (Beyotime, FFNO2, China). After UV crosslinking, the membrane was washed with 1 × PBST buffer, blocked with 5 % non-fat milk, and incubated with m6 A antibody (1:2000, Proteintech, 68055-1, China) overnight at 4 °C. Then, the blots were incubated with the HRP-conjugated Goat Anti-mouse IgG H & L (1:10000, Proteintech, SA00001-1, China) for 1.5 h at room temperature. Then imaged by NcmECL Ultra Reagent A/B (New Cell & Molecular Biotech, P10300, China) and captured by the ChemiDoc MP system (Biorad, 1708280, USA). Methylene blue staining as reference.

### 2.29 RNA immunoprecipitation assay (RIP)

The RNA Binding Protein lmmunoprecipitation Kit (GENE CREATE, JKR23003, China) was used. Briefly, cells were lysed and incubated with magnetic beads conjugated with anti-rabbit METTL14 (5 μg, Proteintech, 26158-1-AP, China), anti-rabbit IGF2BP3 (5 μg, Proteintech, 14642-1-AP, China), or anti-IgG antibodies for more than 8 h at 4 °C. Following the collection of the specific RNA-protein complex that was precipitated from the beads, RNAs were isolated for RT-qPCR analysis.

### 2.30 Cell transfection

The knockdown (sh), overexpression (OE), and matched negative control (NC) plasmids of METTL14 and IGF2BP3 were designed and synthesized by Corues Biotechnology (Nanjing, China). Cell transfection was conducted when the cells achieved 60 %–80 % confluency. All transfections were carried out using Lipofectamine 3000 (Invitrogen, L3000015, USA), following the manufacturer's instructions. The shRNA sequences used were as follows: METTL14 shRNA1: 5′- CCATGTACTTACAAGCCGATA-3′; METTL14 shRNA2: 5′- GCTAATGTT GACATTGACTTA-3′; METTL14 shRNA3: 5′- GCCGTGGACGAGAAAGAAATA-3′; IGF2BP3 shRNA1: 5′-TCTGCGGCTTGTAAGTCTATT-3′; IGF2BP3 shRNA2: 5′-GCAGGAATTGACGCTGTATAA-3′; IGF2BP3 shRNA3: 5′- CGGTGAATGAACTTCAGAATT-3′.

### 2.31 RNA stability assay

The cells were treated with Actinomycin D (Act D, MCE, HY-17559, China) at 10 μg/ml. After incubation for 0 h, 2h, 4h, and 6h, the RNA was extracted, and the expression level of RNA was detected by RT-qPCR as described above. Compared with 0h, the half-life was measured.

### 2.32 Western blot analysis

The total cellular proteins were extracted by RIPA (Yamei, PC101, China) with ProtLytic Protease and Phosphatase Inhibitor Cocktail (1:100, New Cell & Molecular Biotech, P002, China). The protein concentration was detected by BCA Protein Assay Kit (New Cell & Molecular Biotech, WB6501, China), then mixed with SDS-PAGE loading Buffer (5 × , New Cell & Molecular Biotech, WB2001, China) and boiled. The proteins were separated by 10 % ExpressCast PAGE (New Cell & Molecular Biotech, P2012, China) before transferred onto a PVDF membrane (0.45 mm, Merck millipore, IPVH00010, Germany), then incubated with the primary antibodies: METTL14 (1:5000, Proteintech, 26158-1-AP, China), IGF2BP3 (1:6000, Proteintech, 14642-1-AP, China), YAP1 (1:6000, ABclonal, A1002, China), and β-actin (1:7000, Proteintech, 20536-1-AP, China). Then incubated with secondary antibody HRP-conjugated Goat Anti-rabbit IgG H&L (1:10000, Proteintech, SA00001-2, China) or the HRP-conjugated Goat Anti-mouse IgG H&L (1:10000, Proteintech, SA00001-1, China) for 1.5 h, and imaged by NcmECL Ultra Reagent A/B (New Cell & Molecular Biotech, P10300, China) and captured by the ChemiDoc MP system (Biorad, 1708280, USA). Gray value of the band was measured by Image J.

### 2.33 Statistical analysis

This study’ s statistical analysis and data visualization were conducted using GraphPad Prism 9.3, IBM SPSS 22.0, and Image J. Quantitative data are expressed as mean values with standard errors from at least three independent experiments. Comparisons between two data groups were performed using the student’ s t-test, while differences among multiple groups were evaluated using two-way ANOVA with p values. A significance level of p < 0.05 was considered statistically significant. P-values were calculated based on two-sided statistical tests, with significance levels denoted as ∗ p < 0.05, ∗∗p < 0.01, and ∗∗∗p < 0.001. Results with p > 0.05 were considered not significant (ns).

## 3 Results

### 3.1 The spatial transcriptomics and single-cell transcriptomics indicates mechano-glycolytic activation of CSCs within PDAC niche

**Fig. 1:**
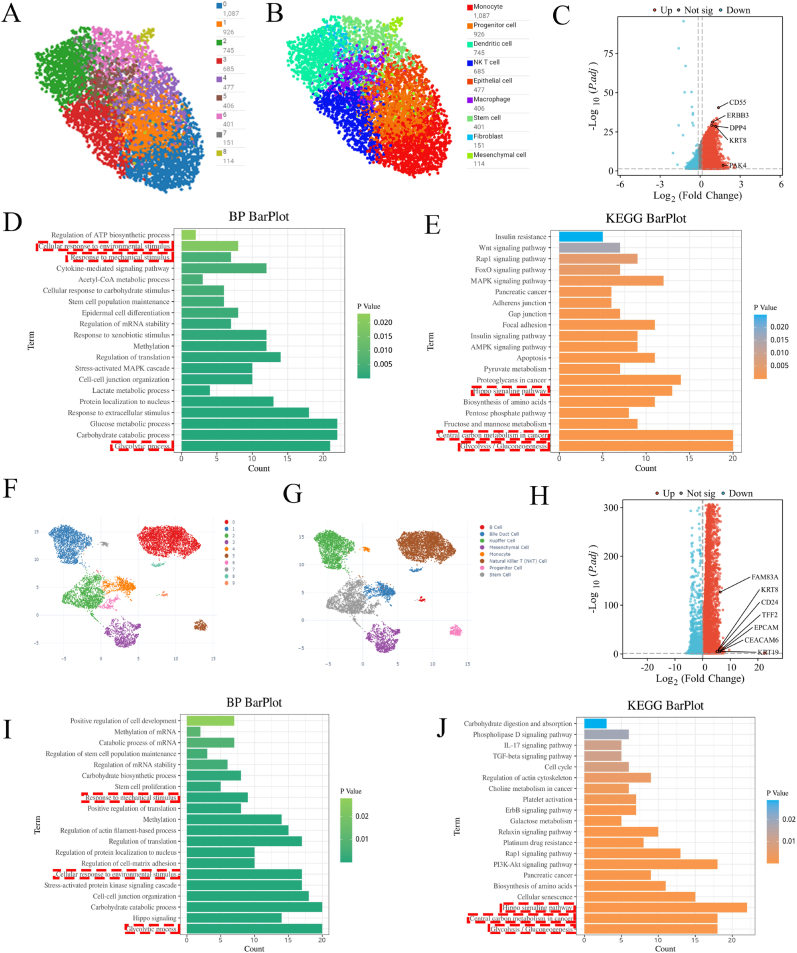
**Mechanical stimulus and glycolysis activation in CSCs within PDAC primary and liver metastatic lesions. (A)** Spatial transcriptomic mapping of primary PDAC lesion (GSE205354) according to the STOmicsDB platform**. (B)** Nine major cell clusters were manually annotated. **(C)** The volcano plot of differentially expressed genes (DEGs) of CSCs (red: upregulated, blue: downregulated, following the STOmicsDB platform default standards) in the primary PDAC lesion. **(D)** Corresponding Gene Ontology (GO) analysis reveals the enriched biological process (BP), including cellular response to the environmental stimulus, response to mechanical stimulus, and glycolytic process (p < 0.05). **(E)** Kyoto Encyclopedia of Genes and Genomes (KEGG) enrichment displays the glycolytic/gluconeogenesis, central carbon metabolism in cancer, and Hippo signaling pathway activation (p < 0.05). **(F)** Single-cell transcriptomic atlas of PDAC liver metastasis lesion (GSE197177) with cluster annotation **(G)** based on the Cell-omics Data Coordinate Platform. **(H)** The volcano plot based on DEGs (red: upregulated, blue: downregulated, according to the Cell-omics Data Coordinate Platform default standards) of CSCs in PDAC liver metastatic lesion. **(I)** Corresponding GO analysis also exhibits the enrichment of cellular response to the environmental stimulus, response to mechanical stimulus, and glycolytic process (p < 0.05). **(J)** KEGG enrichment hints the amplified glycolytic/gluconeogenesis, central carbon metabolism in cancer, and Hippo signaling pathway (p < 0.05). (For interpretation of the references to color in this figure legend, the reader is referred to the Web version of this article.)

Based on the STOmicsDB platform default standards, the spatial transcriptomics dataset of the PDAC primary lesion (GSE205354) was exploited to generate the overall spatial cell atlas (Fig. 1A). Nine major cell clusters were displayed and manually annotated (Fig. 1B) according to the marker gene. The stem cell cluster was identified as the cancer stem cells (CSCs) by upregulating the CSCs markers, such as CD55, ERBB3, KRT8, in the volcano plot (Fig. 1C) based on the differentially expressed genes (DEGs, following the STOmicsDB platform default standards) of the stem cell. There were 5850 DEGs with 5293 upregulated genes and 557 downregulated genes. The Gene Ontology (GO) analysis and Kyoto Encyclopedia of Genes and Genomes (KEGG) analysis were exploited to explore the enriched biological process (BP) and activated signal pathways in CSCs within the primary PDAC lesion. The GO analysis (Fig. 1D) revealed several enriched BP, including cellular response to the environmental stimulus, response to mechanical stimulus, and glycolytic process (p < 0.05). The KEGG analysis (Fig. 1E) revealed the enrichment of glycolytic/gluconeogenesis, central carbon metabolism in cancer, and the Hippo signaling pathway (p < 0.05). We further explore the cell clusters within PDAC liver metastasis lesions by analyzing the single-cell transcriptomics dataset (GSE197177). According to the Cell-omics Data Coordinate Platform default standards, the overall cell clusters (Fig. 1F), predicted cell subtypes (Fig. 1G), and the volcano plot of stem cell DEGs (Fig. 1H) were obtained. The stem cell cluster within the PDAC liver metastasis lesion exhibited high expression of FAM83A, KRT8, CD24, TFF2, and other CSC markers in the volcano plot of DEGs (Fig. 1H) and was identified as CSCs. The CSCs within the PDAC liver metastasis lesion exhibited 9064 upregulated genes and 2738 downregulated genes. The corresponding GO analysis (Fig. 1I) shows the shared BP enriched in the CSCs within the PDAC primary lesion, including the cellular response to the environmental stimulus, response to mechanical stimulus, and glycolytic process (p < 0.05). It is noteworthy that PDAC is one of the stiffest solid tumors with a unique desmoplastic tumor microenvironment (TME), which indicates that the stimulus, especially the mechanical stimulus, provided by the PDAC TME, exerts a key role in the CSCs. The KEGG analysis (Fig. 1J) displayed the enriched glycolytic/gluconeogenesis, central carbon metabolism in cancer, and the Hippo signaling pathway (p < 0.05), which were also activated in the CSCs in the PDAC primary lesion. The Hippo signaling pathway is involved in mechanotransduction, further prominent the role of mechanical stimulus in CSCs. Glycolysis, the core component of central carbon metabolism, emerged in GO and KEGG analysis of the CSCs DEGs within both the PDAC primary and live metastasis lesions. Collectively, these may position mechanical stress and metabolic reprogramming as dual regulators of PDAC stem-like phenotype.

### 3.2 Spatial stiffness heterogeneity in human primary PDAC

**Fig. 2:**
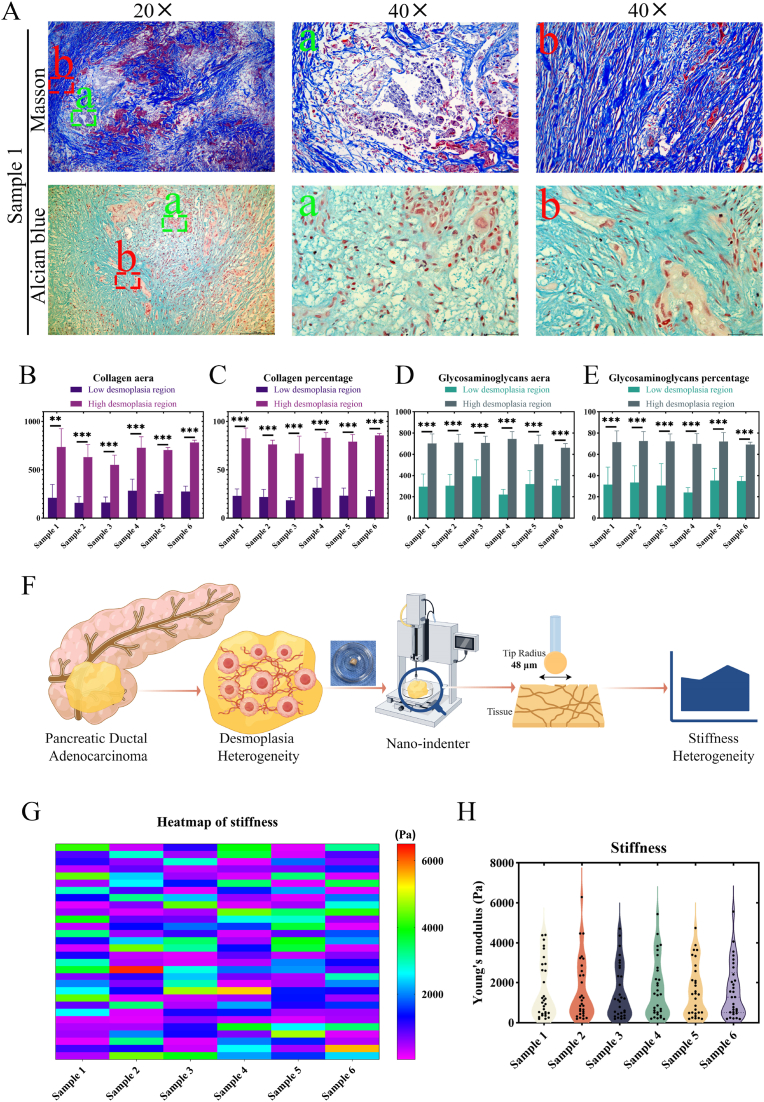
**Stromal and biomechanical heterogeneity within the human primary PDAC tissues. (A)** Representative Masson’ s trichrome (collagen, blue) and Alcian blue (glycosaminoglycans, GAGs, turquoise) staining images of PDAC tissues (Sample 1). Local regions with low (green dashed, a) and high (red dashed, b) desmoplastic stroma are indicated; scale bar: 200 μm (overview), 50 μm (inset). The area and percentage quantification of collagen **(B**–**C)** and GAGs **(D**–**E)** in low and high desmoplastic local regions within PDAC tissues (n = 3, p < 0.05). **(F)** Schematic of nanoindentation-based stiffness mapping for PDAC tissues, created by Figdraw. **(G)** Stiffness heatmap of PDAC tissues measured by nanoindentation (50 points per sample, n = 6). **(H)** Violin diagram of the Young’ s modulus value in the local region of PDAC, ranging from about 200 to 6000 Pa. Data: mean ± SD, ∗p < 0.05, ∗∗p < 0.01, ∗∗∗p < 0.001, ∗∗∗∗p < 0.0001, and n.s.: denotes not significant. (For interpretation of the references to color in this figure legend, the reader is referred to the Web version of this article.)

PDAC is typically characterized by a dense extracellular matrix (ECM, desmoplasia), which can constitute up to 90 % of the tumor tissue and has been assumed to become stiffer as a whole in most previous studies. And the enrichment, deposition, and crosslinking of ECM components, mainly the collagen and glycosaminoglycans (GAGs), resulted in an abnormal increase in TME stiffness [24]. The Masson’ s Trichrome and Alcian blue (Fig. 2A and S-Fig. 1A-B) were further stained in the human primary PDAC tissues (n = 6) to detect the content of collagen (blue) and GAGs (turquoise). Notably, the results displayed the inhomogeneous enrichment and deposition of collagen and GAGs. The dense region is defined as high local desmoplasia, while the loose region is low local desmoplasia. Quantitative analysis of collagen area (Fig. 2B) and percentage (Fig. 2C) in high local desmoplastic regions is significantly higher than in the low local regions (n = 3, p < 0.05). The same conclusion also occurs in the quantitation of GAGs (Fig. 2D–E, n = 3, p < 0.05). These strongly indicate that there is heterogeneity of local stiffness within the PDAC niche and prompt us to determine the local stiffness spectrum by nano-indenter (Fig. 2F). The heatmap based on Young’ s modulus intuitively reflects the local niche stiffness heterogeneity (Fig. 2G). Quantitative analysis reveals the stiffness variations spanning 200-6000 Pa (Fig. 2H). This stiffness spectrum mirrors intra-tumoral stiffness heterogeneity patterns that have been reported in breast cancer [42]. This local niche biomechanical diversity, induced by significant intra-tumoral heterogeneity, which is the hallmark of cancer [13]. These establish PDAC as a mechanically graded ecosystem with underexplored cellular consequences.

### 3.3 Digital light-processing (DLP) 3D-printing stiffness-graded PDAC niches matching tumor biomechanical heterogeneity

**Fig. 3:**
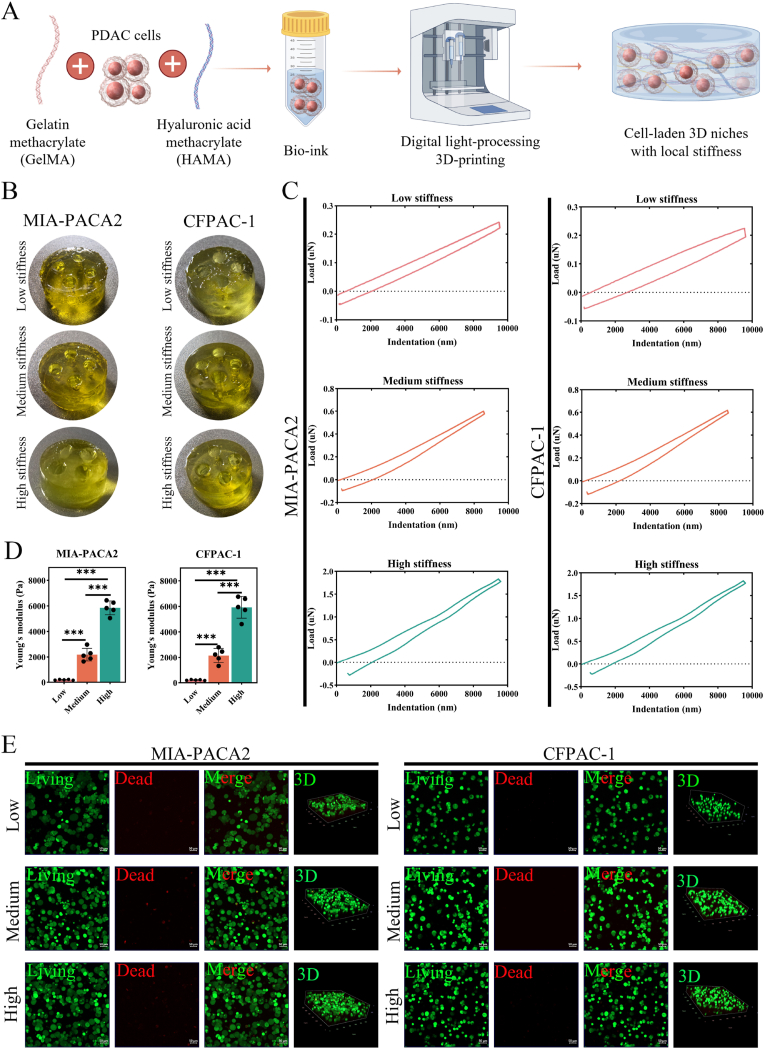
**A Digital light-processing (DLP) 3D-printing biomimetic local stiffness niches simulate PDAC biomechanical heterogeneity. (A)** Schematic of digital light-processing-based 3D bioprinting for fabricating PDAC-mimetic local stiffness niche, created by Figdraw. Gelatin methacrylate (GelMA) and hyaluronic acid methacrylate (HAMA) replicate extracellular matrix (ECM) components in the desmoplastic tumor microenvironment (TME). Stiffness is modulated via crosslinking density dependent on photocuring time while maintaining constant bioink composition. **(B)** Macrograph of 3D-printed biomimetic local stiffness PDAC niches encapsulated with MIA-PACA2 and CFPAC-1 cells and the corresponding load-displacement curves **(C)**. **(D)** Young’ s modulus of the biomimetic niches containing MIA-PACA2 and CFPAC-1 cells with low (193.1 ± 26.89 Pa and 212.9 ± 31.74 Pa), medium (2174 ± 506.9 Pa and 2145 ± 563.6 Pa), and high local stiffness (5848 ± 543.2 Pa and 5926 ± 851.3 Pa) (n = 5, p < 0.05). **(E)** Viability of PDAC cells after 5-day culture in the niches with the low, medium, and high local stiffness (calcein-AM/PI, green: live; red: dead; scale bar: 50 μm). Data: mean ± SD, ∗p < 0.05, ∗∗p < 0.01, ∗∗∗p < 0.001, ∗∗∗∗p < 0.0001, and n.s.: denotes not significant. (For interpretation of the references to color in this figure legend, the reader is referred to the Web version of this article.)

3D printing offers the potential to accurately deposit biomaterials and cells, enabling the creation of heterogeneous tissue-mimetic constructs that enhance cell-ECM crosstalk within a 3D setting, which are lacking in traditional 2D cell culture systems [43]. This significant advancement has been widely applied in various cancer research studies to simulate the traits of the TME. Furthermore, progressing towards the 3D bioprinting of stromal elements is an essential step in developing next-generation tumor models [44]. And in our previous study, the dual-crosslinked 7.5 % (w/v) GelMA &1 % (w/v) HAMA hydrogel system was developed to construct the PDAC desmoplastic niche, which featured with mimicking the main component and the crosslinking state of ECM. In addition, its mechanical properties could be adjusted by ultraviolet light (UV) cross-linking duration without changing the concentration. These inspired us to exploit it as cells-laden bioink to construct stiffness-tunable niches matching the local niche stiffness heterogeneity within PDAC based on digital light-processing (DLP) 3D-printing (Fig. 3A). We first analyzed the microstructure of cell-free hydrogels with varying stiffness using a scanning electron microscope (SEM). All hydrogels group displayed a porous structure (S-Fig. 2A), which enhanced cell attachment, infiltration, and material transport. And the porosity without significant difference (S-Fig. 2B, n = 3, p > 0.05). Then, the PDAC cells (MIA-PACA2 and CFPAC-1 cells)-encapsulated bioink exhibited expected rheology behavior (S-Fig. 2C) with storage modulus (G′, elastic component) much greater than loss modulus (G″, viscous component) and characteristic shear-thinning behavior (S-Fig. 2D), which are crucial for micro-extrusion and gelling in 3D printing. Further, we construct the 3D printing biomimetic local stiffness niches with low, medium, and high stiffness (Fig. 3B). And corresponding modeling diagram by CAD were displayed in S-Fig. 2E. With the load-displacement curves based on the nano-indenter detection (Fig. 3C), the Young’ s modulus for encapsulated MIA-PACA2 cells niche with the low, medium, and high local stiffness is approximately 193.1 ± 26.9 Pa, 2174.0 ± 506.9 Pa, and 5848.0 ± 543.2 Pa, respectively. And for the CFPAC-1 cells niche is approximately 212.9 ± 31.7 Pa, 2145.0 ± 563.6 Pa, and 5926.0 ± 851.3 Pa, respectively. The quantitative analysis of Young’ s modulus reveals that there is a significant difference among the low, medium, and high local stiffness groups (Fig. 3D, n = 5, p < 0.05). And the local stiffness within biomimetic niches also exhibited significantly different and covers the minimum, medium, and maximum Young’ s modulus of the PDAC intratumor heterogeneous stiffness spectrum (S-Fig. 2F, n = 5, p < 0.05). The swelling behavior was assessed, and biomimetic niches across local stiffness groups reached equilibrium swelling after approximately 12 h (S-Fig. 2G). Thers is no significant difference in swelling ratio (S-Fig. 2I, n = 5, p > 0.05), for biomimetic local niches containing MIA-PACA2 and CFPAC-1 cells with low (995.3 ± 22.9 % and 1006.0 ± 31.2 %), medium (966.2 ± 36.03 % and 873.5 ± 35.9 %), and high stiffness (939.2 ± 49.3 % and 943.9 ± 53.2 %) group. These also suggest a high porosity of the hydrogel network, resulting in a good level of medium diffusion within the gel [45,46]. We further conducted the degradation test (S-Fig. 2H). The results showed that the weight ratio in the low, medium, and high local stiffness biomimetic local niches encapsulated MIA-PACA2 cells on day 12 is approximately 85.2 ± 2.9 %, 85.5 ± 2.7 %, 85.8 ± 3.5 %, respectively. And for the CFPAC-1 cells niche is approximately 83.9 ± 3.1 %, 84.6 ± 2.0 %, and 85.6 ± 0.8 %, respectively. And quantitation with no significant difference (S-Fig. 2J, n = 5, p > 0.05). These indicate that the hydrogel has good stability during culture. After culturing for 5 days, live/dead staining (Fig. 3E) revealed that encapsulated PDAC cells exhibited great viability with no significant viability difference across stiffness conditions (S-Fig. 2K, n = 5, p > 0.05). H&E staining showing the matrix architecture and uniform distribution of PDAC cells across the groups (S-Fig. 2L–M). Collectively, the 3D-printed biomimetic local stiffness niches possess favorable biocompatibility and greatly simulate heterogeneous PDAC stiffness, providing a physio-mimetic platform for mechanobiology studies.

### 3.4 Local stiff niches-driven stem-like phenotype via glycolytic reprogramming

**Fig. 4:**
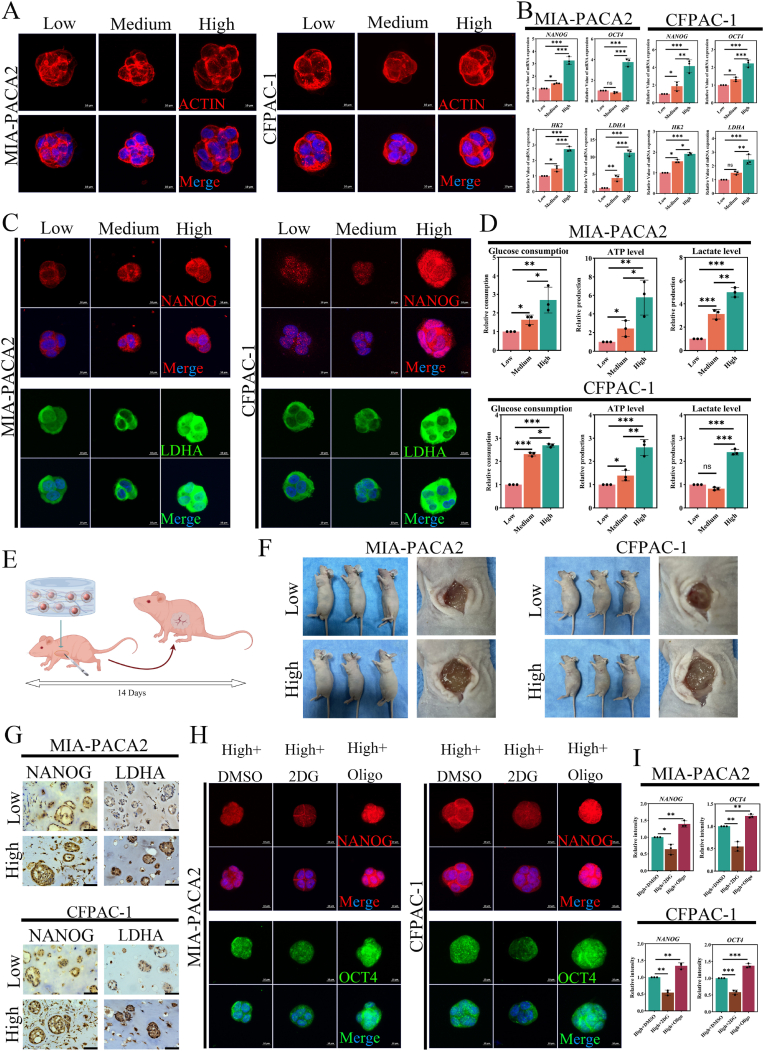
**High local stiffness-driven glycolytic reprogramming potentiates PDAC cell stem-like phenotype. (A)** PDAC cells exhibited 3D spheroids morphology across the local stiffness group (Phalloidin: red, scale bar: 10 μm). **(B)** Upregulation of stemness markers (NANOG, OCT4) and glycolysis markers (HK2, LDHA) in high local stiffness niches (qRT-PCR, n = 3, p < 0.05, vs low local stiffness niches). **(C)** Representative immunofluorescence of NANOG (red) and LDHA (green) for PDAC cells within across local stiffness group, scale bar: 10 μm. **(D)** Quantitative analysis showing relative glucose consumption, ATP production, and lactate production of PDAC cells are significant boosted in high local stiffness group (n = 3, p < 0.05, vs low local stiffness group). **(E)** A flowchart depicting the process of establishing subcutaneous 3D-printed models of PDAC in nude mice, created by Figdraw. **(F)** Images of mice at the time of sacrifice (n = 3). **(G)** Representative immunohistochemistry images of NANOG and LDHA in subcutaneously transplanted 3D printed PDAC niches (low local and high local stiffness group, scale bar: 50 μm). **(H)** Representative immunofluorescence characterization of NANOG (red) and OCT4 (green) of PDAC cells within the high local stiffness niches treated with 5 mM 2-Deoxy-D-glucose (2DG) for 12 h or 5 μM oligomycin (Oligo) for 12 h, scale bar: 10 μm. **(I)** Quantification of NANOG and OCT4 fluorescence intensity in high local stiffness group treated with 2DG or Oligo (n = 3, p < 0.05, vs high local stiffness + DMSO group), Data: mean ± SD, ∗p < 0.05, ∗∗p < 0.01, ∗∗∗p < 0.001, ∗∗∗∗p < 0.0001, and n.s.: denotes not significant. (For interpretation of the references to color in this figure legend, the reader is referred to the Web version of this article.)

After culturing for 5 days, the representative cellular morphology via z-stack confocal was shown in Fig. 4A, and PDAC cells exhibited a multi-cellular spheroid stereoscopic structure in the biomimetic local niches with low, medium, and high stiffness. The biological process of CSCs within PDAC exhibited activation of mechanical stimulus and glycolysis (Fig. 1D and I). And nanog homeobox (NANOG) and pou class 5 homeobox 1 (OCT4), involved in maintaining the stemness of PDAC CSCs in our previous study [47], are identified as stem-like phenotype markers. Hexokinase 2 (HK2) and lactate dehydrogenase A (LDHA), the key enzymes involved in the glycolysis process, are exploited as glycolysis markers. We evaluated the expression levels of NANOG, OCT4, HK2, and LDHA of 3D printing biomimetic local niches with low, medium, and high stiffness by qRT-PCR (Fig. 4B) and immunofluorescence (Fig. 4C and S-Fig. 3A). High local stiffness niche markedly upregulated NANOG, OCT4, HK2, and LDHA at the transcriptional level (n = 3, p < 0.05), which is consistent with the quantitative analysis of the immunofluorescence intensity (S-Fig. 3B, n = 3, p < 0.05). The stemness of cancer cells contributes to their resistance to chemotherapy. To further investigate this, we examined the sensitivity of PDAC cells to gemcitabine (GEM, S-Fig. 3C), which is the first-line chemotherapy agent for PDAC. For MIA-PACA2 cells, the half maximal inhibitory concentration (IC_50_) values for GEM based on the dose-response curves were approximately 14.4 ± 6.2 μM in the low stiffness group, 22.9 ± 6.5 μM in the medium stiffness group, and 52.7 ± 12.3 μM in the high stiffness group (n = 5). For CFPAC-1 cells, the IC_50_ values were approximately 9.5 ± 1.7 μM, 16.5 ± 3.0 μM, and 39.3 ± 4.8 μM, respectively (n = 5). Additionally, the efficiency of cell spheroid formation significantly increased with higher local stiffness (S-Fig. 3D–E, n = 3, p < 0.05). Functional assays confirmed stiffness-dependent glycolysis shifts, with high local stiffness niche elevating glucose consumption, ATP production, and lactate production versus the low local stiffness niche (Fig. 4D, n = 3, p < 0.05). Moreover, 5-week BALB/c nude mice received subcutaneous implants of 3D-printed niches (Fig. 4E–F). The immunohistochemistry also revealed that high local stiffness enhanced the level of NANOG, OCT4, HK2, and LDHA in vivo (Fig. 4G and S-Fig. 3F). These collaborations prove that high local stiffness supports the PDAC stem-like cell phenotype and glycolysis. And metabolic reprogramming was recognized as one of the hallmarks of cancer cells. Meanwhile, Lanfranca et al. proposes that CSCs exhibit metabolic plasticity that can adjust to the settings in which they reside [48]. Namely, the high local stiffness in PDAC may boost the stem-like phenotype via glycolysis augmentation. The rescue experiment with 2-Deoxy-D-glucose (2DG, 5 mM, 12 h, glycolysis inhibitor) and oligomycin (Oligo, 5 μM, 12 h, promotes compensatory glycolysis) was carried out. The results of qRT-PCR demonstrate that 2DG significantly attenuated high local stiffness-induced NANOG and OCT4 levels compared to the control, while the Oligo exerted a converse tendency (S-Fig. 3G, n = 3, p < 0.05). The corresponding immunofluorescence and quantitative analysis yielded the same conclusion (Fig. 4H–I, n = 3, p < 0.05). As shown in S-Fig. 3H, for MIA-PACA2 cells, the IC_50_ values of GEM were approximately 51.2 ± 9.9 μM in the high + DMSO group, 19.9 ± 2.6 μM in the high + 2DG group, and 94.9 ± 17.9 μM in the high + Oligo group (n = 5). For CFPAC-1 cells, the IC_50_ values of rescue experiments were approximately 36.7 ± 5.1 μM, 17.5 ± 3.9 μM, and 63.3 ± μ10.0 μM, respectively (n = 5). Meanwhile, the efficiency of cell spheroid formation of the high + 2DG group significantly decreased, while the high + Oligo group considerably increased, compared to the high + DMSO group (S-Fig. 3I–J, n = 3, p < 0.05). These demonstrated and established the mechano-metabolic crosstalk in PDAC cells stem-like phenotype regulation, while the potential mechanism still needs to be elucidated.

### 3.5 YAP1 predicts poor prognosis and is boosted by local stiff niche in PDAC

**Fig. 5:**
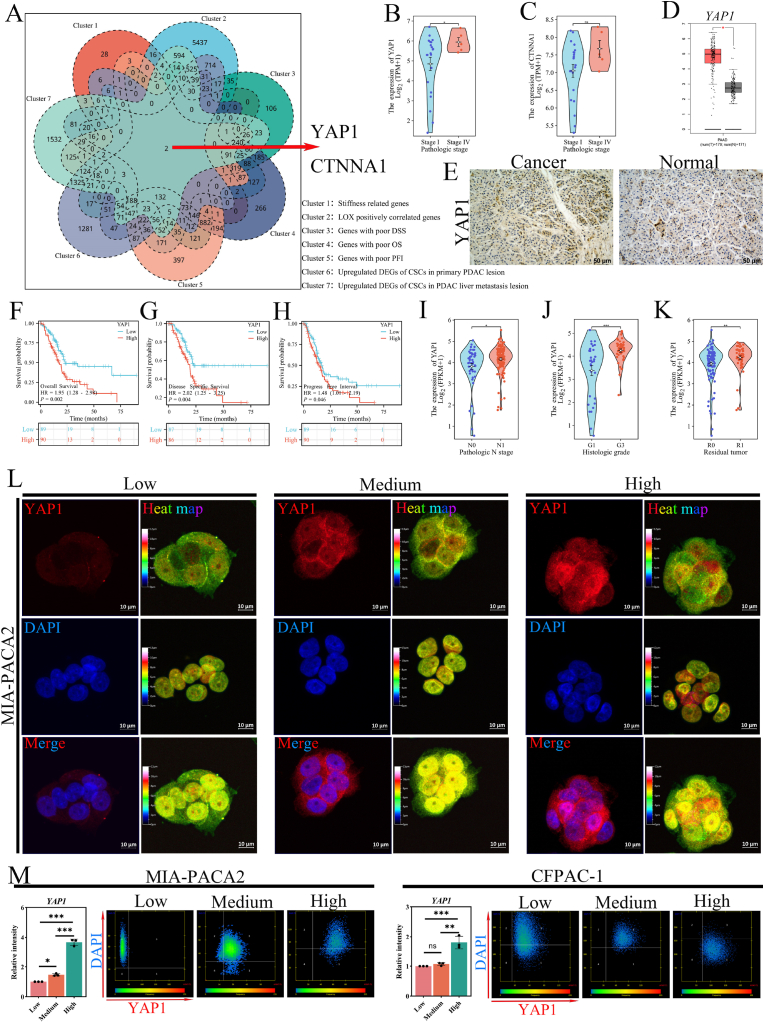
**YAP1 is closely linked to tumor stiffness and predicts poor prognosis in PDAC. (A)** Overlapping analysis identifies YAP1 as a core regulator intersecting stiffness-associated genes (GeneCards top 100), LOX positively correlated genes (R > 0.145, p < 0.05, TCGA-PAAD), poor survival predictors in PDAC (DSS/OS/PFI, p < 0.05, TCGA-PAAD), and the upregulated DEGs (following the analysis platform default standards) of CSCs within primary PDAC lesion (GSE205354) and liver metastasis lesion (GSE197177). **(B**–**C)** The expression of YAP1 and CTNNA1 in the PDAC tissue IV stage vs I stage (AJCC 8th tumor stage, TCGA-PAAD). **(D)** Elevated YAP1 expression in PDAC tissue vs the adjacent (p < 0.05, GEPIA2). **(E)** Representative YAP1 immunohistochemistry in PDAC and normal pancreas (scale bar: 50 μm). **(F**–**H)** Kaplan-Meier survival curves showing reduced OS, DSS, and PFI in YAP1-high patients (p < 0.05, TCGA-PAAD). **(I**–**K)** Violin plots illustrate the positive relationships between YAP1 expression and N stage, G histologic stage, and residual tumor state. R0 resection indicates a complete resection where no residual tumor is found at the microscopic level. R1 resection means that there is microscopic residual tumor at the resection margins. **(L)** Representative immunofluorescence images and heatmap of YAP1 (red) in MIA-PACA2 cells across the local stiffness group (scale bar:10 μm). **(M)** The quantification of fluorescence intensity displays that YAP1 is elevated in the high local stiffness group significantly (n = 3, p < 0.05, vs low local stiffness group), and correlation analysis confirms nuclear YAP1 accumulation correlates with increased local stiffness. Data: mean ± SD, ∗p < 0.05, ∗∗p < 0.01, ∗∗∗p < 0.001, ∗∗∗∗p < 0.0001; n.s.: not significant. (For interpretation of the references to color in this figure legend, the reader is referred to the Web version of this article.)

Lysyl-oxidase (LOX) plays a significant role in cancer-associated ECM stiffness by crosslinking ECM components [49]. The LOX level is significantly higher in PDAC tissues than in the normal, and high LOX expression predicted poor overall survival (OS), disease specific survival (DSS), and progress free interval (PFI, S-Fig. 4A–D, p < 0.05). To investigate the key genes that boost the stem-like phenotype of PDAC cells in response to the mechanical cues, we overlapped genes from stiffness-associated genes top 100 (GeneCards), LOX positively correlated genes in PDAC (R > 0.145, p < 0.05, TCGA-PAAD), genes predicated poor DSS/OS/PFI in PDAC (p < 0.05, TCGA-PAAD), and upregulated DEGs (following the analysis platform default standards) of CSCs within primary PDAC lesion (GSE205354) and PDAC liver metastasis lesion (GSE197177). Two genes, YAP1 and CTNNA1, emerged (Fig. 5A). Zhang et al. reveal that the collagen content (as the ECM stiffness marker of PDAC [50]) was positively correlated with the increased stage (AJCC 8th tumor stage) [51]. YAP1 in IV stage patients is remarkably elevated compared to the I stage (Fig. 5B, p < 0.05), while CTNNA1 is not significant (Fig. 5C, p > 0.05). Hence, YAP1 is selected for subsequent research. In PDAC, YAP1 is not only significantly positively co-expressed with LOX (S-Fig. 4E, R = 0.87, p < 0.05), but also highly expressed in LOX-high group patients compared to the low group patients (S-Fig. 4F, p < 0.05). Further, YAP1 is overexpressed in the PDAC tissues than the normal tissues based on the GEPIA database (Fig. 5D, p < 0.05) and immunohistochemistry (Fig. 5E and S-Fig. 4G). High YAP1 expression predicted poor OS, DSS, and PFI (Fig. 5F–H, p < 0.05), and the 5-year area under the curve (AUC) = 0.772, 0.778, and 0.898, respectively (S-Fig. 4H–J). Additionally, the level of YAP1 level was positively correlated with the N stage (Fig. 5I, N1 vs N0, p < 0.05), G histologic stage (Fig. 5J, G3 vs G1, p < 0.05), and R residual tumor state (Fig. 5K, R1 vs R0, p < 0.05). Meanwhile, clinical and pathological data were summarized in Table 1, which indicated that the YAP1 overexpression was an independent predictor of histologic stage, pathologic stage, the chronic pancreatitis history, OS, and DSS in PDAC patients. Chronic pancreatitis is characterized by stromal fibrosis and is considered a risk factor for PDAC. To explore the role of YAP1 in response to the heterogeneous stiffness within PDAC, qRT-PCR analysis (S-Fig. 5A) and immunofluorescence (Fig. 5L and S-Fig. 5B) were performed. The quantitative analysis of immunofluorescence intensity shows that the level of YAP1 was significantly elevated in the high local stiffness group (Fig. 5M, n = 3, p < 0.05), which is consistent with the result of the qRT-PCR (n = 3, p < 0.05). In addition, representative immunohistochemistry images also show the upregulated expression of YAP1 in the high local stiffness niches in vivo (S-Fig. 5C). As a transcriptional coactivator, YAP1 transitions from the cytoplasm to the nucleus, where it interacts with TEAD and other transcription factors to stimulate gene expression. We further detected its subcellular localization based on the confocal microscope. The heat map of immunofluorescence (Fig. 5L and S-Fig. 5B) and the correlation analysis of DAPI and YAP1 (Fig. 5M) demonstrated that the high local stiffness promotes YAP1 aggregation in the nucleus. These hints at the potential role of YAP1 for PDAC cells in response to the heterogeneous stiffness within TME.

**Table 1:** Patient characteristics and comparison between low YAP1 and high YAP1.

Characteristics	Low expression of YAP1	High expression of YAP1	p value
n	89	90	
Gender, n (%)			0.504
Female	42 (23.5 %)	38 (21.2 %)	
Male	47 (26.3 %)	52 (29.1 %)	
Age, n (%)			0.086
≤ 65	41 (22.9 %)	53 (29.6 %)	
>65	48 (26.8 %)	37 (20.7 %)	
History of diabetes, n (%)			0.368
No	58 (39.5 %)	51 (34.7 %)	
Yes	17 (11.6 %)	21 (14.3 %)	
Alcohol history, n (%)			0.591
No	31 (18.6 %)	34 (20.4 %)	
Yes	53 (31.7 %)	49 (29.3 %)	
Family history of cancer, n (%)			0.297
No	21 (18.9 %)	26 (23.4 %)	
Yes	35 (31.5 %)	29 (26.1 %)	
History of chronic pancreatitis, n (%)			**0.037**
No	69 (48.6 %)	60 (42.3 %)	
Yes	3 (2.1 %)	10 (7 %)	
Smoker, n (%)			0.050
No	40 (27.6 %)	26 (17.9 %)	
Yes	35 (24.1 %)	44 (30.3 %)	
Pathologic T stage, n (%)			0.074
T1	3 (1.7 %)	4 (2.3 %)	
T2	16 (9 %)	8 (4.5 %)	
T3	65 (36.7 %)	78 (44.1 %)	
T4	3 (1.7 %)	0 (0 %)	
Pathologic N stage, n (%)			0.062
N0	30 (17.2 %)	20 (11.5 %)	
N1	55 (31.6 %)	69 (39.7 %)	
Pathologic M stage, n (%)			1.000
M0	35 (41.2 %)	45 (52.9 %)	
M1	2 (2.4 %)	3 (3.5 %)	
Histologic grade, n (%)			**0.021**
G1	23 (13 %)	8 (4.5 %)	
G2	45 (25.4 %)	51 (28.8 %)	
G3	19 (10.7 %)	29 (16.4 %)	
G4	1 (0.6 %)	1 (0.6 %)	
Pathologic stage, n (%)			**0.037**
Stage I	15 (8.5 %)	6 (3.4 %)	
Stage II	66 (37.5 %)	81 (46 %)	
Stage III	3 (1.7 %)	0 (0 %)	
Stage IV	2 (1.1 %)	3 (1.7 %)	
Residual tumor, n (%)			0.321
R0	55 (33.3 %)	52 (31.5 %)	
R1	21 (12.7 %)	32 (19.4 %)	
R2	3 (1.8 %)	2 (1.2 %)	
Primary therapy outcome, n (%)			0.187
PD&PR	24 (17.1 %)	36 (25.7 %)	
SD&CR	41 (29.3 %)	39 (27.9 %)	
Radiation therapy, n (%)			0.301
No	58 (35.4 %)	61 (37.2 %)	
Yes	26 (15.9 %)	19 (11.6 %)	
OS event, n (%)			**< 0.001**
Alive	54 (30.2 %)	32 (17.9 %)	
Dead	35 (19.6 %)	58 (32.4 %)	
DSS event, n (%)			**0.003**
No	60 (34.7 %)	40 (23.1 %)	
Yes	27 (15.6 %)	46 (26.6 %)	
PFI event, n (%)			0.060
No	43 (24 %)	31 (17.3 %)	
Yes	46 (25.7 %)	59 (33 %)	

**Table Notes:** This table comprehensively presents the demographic, clinical, and histopathologic characteristics of all patients (N = 179), stratified by YAP1 levels into the low YAP1 group (N = 89) and the high YAP1 group (N = 90). Categorical variables were compared using Chi-square tests, while continuous variables were analyzed with student's t-tests (or suitable non-parametric tests, where applicable, when normality assumptions were violated). Pathologic T, Pathologic N, and Pathologic M for pathological staging. G1, G2, and G3 for histological staging. In this study, a p-value less than 0.05 was considered statistically significant and was highlighted in bold.

### 3.6 High local stiffness-induced YAP1 reprograms glycolytic metabolism in PDAC

**Fig. 6:**
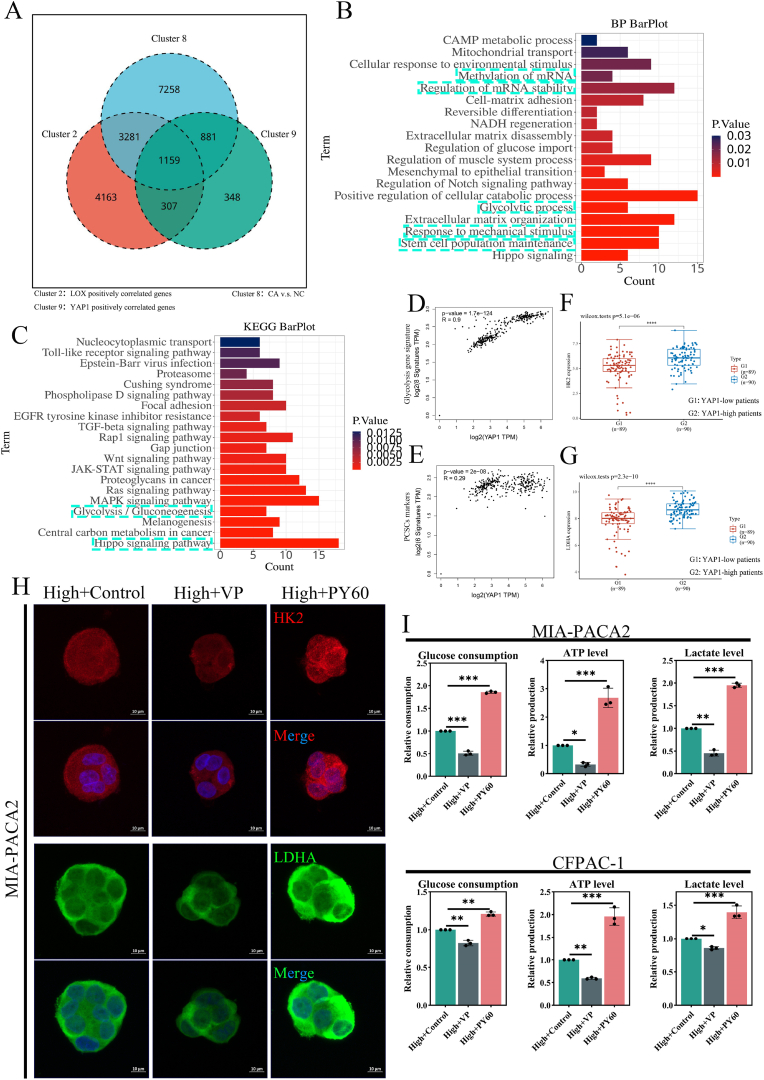
**YAP1 mediates high local stiffness-dependent glycolytic reprogramming in PDAC. (A)** Venn of overlapping upregulated DEGs (CA vs NC, log2 fold change >0.58, p value < 0.05, TCGA-PAAD-GTEx), LOX positively correlated genes (R > 0.145, p < 0.05, TCGA-PAAD), and YAP1 positively correlated genes in PDAC (R > 0.145, p < 0.05, TCGA-PAAD). **(B)** GO analysis based on the Venn hints at the enriched biological process, including regulation of mRNA stability, methylation of mRNA, glycolytic processes, response to the mechanical stimulus, and stem cell population maintenance (p < 0.05). **(C)** KEGG pathway analysis highlights Hippo signaling and glycolysis/gluconeogenesis activation. Scatterplot showing the correlation between YAP1 and glycolysis gene signature **(D**, R = 0.90, p < 0.05**)** and PDAC CSCs markers **(E**, R = 0.29, p < 0.05**)** in PDAC patients (GEPIA2). **(F**–**G)** HK2 and LDHA expression in YAP1 high group (n = 90) vs YAP1 low group (n = 89, p < 0.05, TCGA-PAAD). **(H)** Representative immunofluorescence images showing VP-mediated (1 μg/mL, 12 h) suppression and PY60-induced (10 μM, 12 h) recovery of HK2 (red) and LDHA (green) expression for MIA-PACA2 cells in the high local stiffness group (scale bar: 10 μm). **(I)** PY60 enhanced glucose consumption, ATP production, and lactate production for PDAC cells in the high local stiffness niche, while VP showed opposing effects (n = 3, p < 0.05, vs high local stiffness + control group). Data: mean ± SD, ∗p < 0.05, ∗∗p < 0.01, ∗∗∗p < 0.001, ∗∗∗∗p < 0.0001; n.s.: not significant. (For interpretation of the references to color in this figure legend, the reader is referred to the Web version of this article.)

We intersected the upregulated DEGs (CA vs NC, log2 fold change >0.58, p value < 0.05, TCGA-PAAD-GTEx), LOX positively correlated genes (R > 0.145, p < 0.05, TCGA-PAAD), and YAP1 positively correlated genes (R > 0.145, p < 0.05, TCGA-PAAD) in PDAC to obtain the DEGs of mechanical sensitivity related to YAP1 (Fig. 6A), which were further for GO and KEGG analysis. The GO analysis (Fig. 6B) exhibited a significantly enriched biological process of the stem cell population maintenance (p < 0.05), which further highlights the function of stiffness and YAP1 on the stem-like phenotype of PDAC cells. And notably, it also exhibited the shared biological process of PDAC CSCs in primary and liver metastasis lesions (Fig. 1D and I), including response to mechanical stimulus, glycolytic process, and mRNA stability regulation (p < 0.05). Meanwhile, the KEGG (Fig. 6C) analysis showed the enrichment of the Hippo signaling pathway and glycolysis/gluconeogenesis signaling pathway, which are also enriched in PDAC CSCs (Fig. 1E and J, p < 0.05). Moreover, the expression of YAP1 was positively correlated with glycolysis gene signature (including HK2, LDHA, HIF1a, GAPDH, PKM, PFKM, SLC2A2, PGK2) (Fig. 6D, R = 0.90, p < 0.05) and PDAC CSCs markers (NANOG, OCT4, NR5A2, HNF1a, CD44, CD24) (Fig. 6E, R = 0.29, p < 0.05). Meanwhile, the levels of HK2 and LDHA are higher in YAP1-high group patients than in the low group patients (Fig. 6F–G, p < 0.05). We speculate that YAP1 functioned as the chain crosslink of the high local stiffness and glycolysis reprogramming, further boosting the stem-like phenotype. The verteporfin (VP, YAP1 inhibitor) and PY60 (YAP1 activator) were exploited in our research. And the high local stiffness niches were treated with VP (1 μg/mL, 12 h) or PY60 (10 μM, 12 h). The results of qRT-PCR demonstrate that VP suppressed HK2 and LDHA levels compared to the control, while PY60 exerted a converse tendency (S-Fig. 6A, n = 3, p < 0.05). The immunofluorescence and corresponding quantitative analysis yielded the same conclusion (Fig. 6H and S-Fig. 6B-C, n = 3, p < 0.05). Meanwhile, the glucose consumption, ATP production, and lactate production of the high local niches are remarkably amplified with the treatment of PY60, while they are decreased significantly with the treatment of VP compared to the control (Fig. 6I, n = 3, p < 0.05). And we have illustrated that the local stiff niches drive glycolysis to boost the PDAC cell stem-like phenotype mentioned above. It is reasonable to infer that the local stiff niches boost the PDAC cell stem-like phenotype via YAP1-mediated glycolysis.

### 3.7 The local stiff niche up-regulates YAP1 level via METTL14/IGF2BP3-suppressed YAP1 mRNA decay

**Fig. 7:**
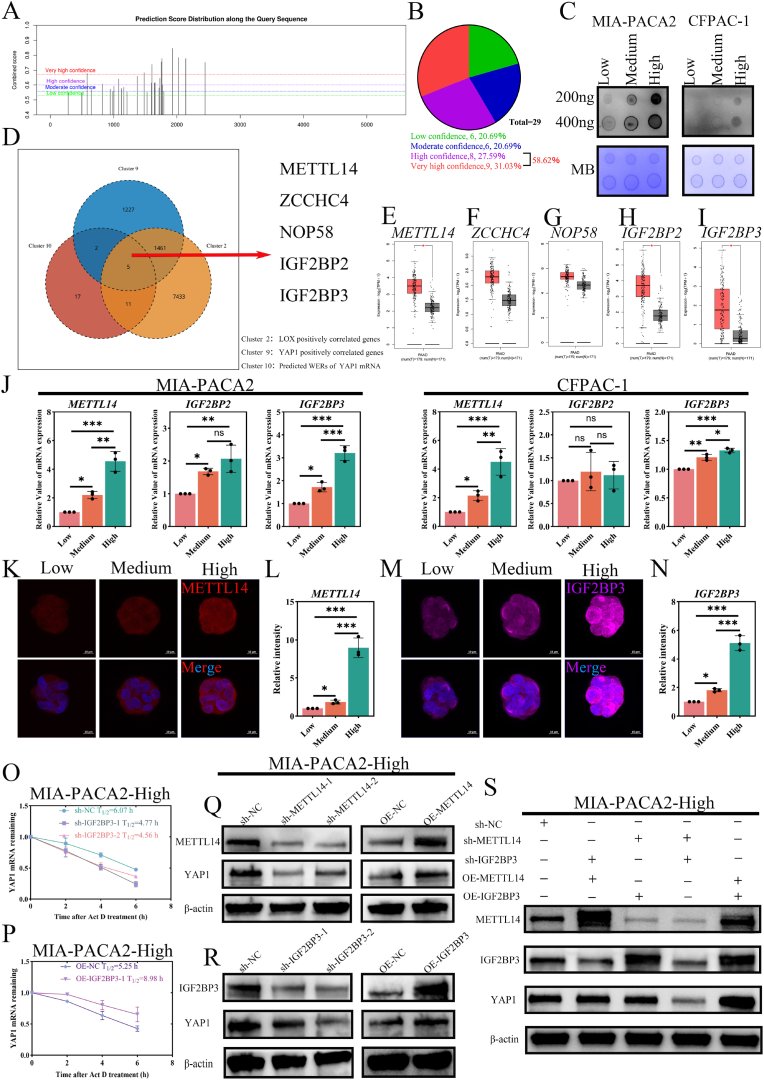
**METTL14/IGF2BP3 respond to the high local stiffness suppressed the decay of YAP1 mRNA. (A**–**B)** Predicted m6A motifs on YAP1 mRNA (SRAMP database, red: very high confidence, purple: high confidence). **(C)** Global m6A elevation in low, medium, and high local stiffness niches (Dot blot). **(D)** Overlap analysis of stiffness-responsive m6A WERs (writers, erasers, readers) targeting YAP1 by intersecting LOX and YAP1-correlated genes (R > 0.145, p < 0.05, TCGA-PAAD) and YAP1-predicted WERs (RM2Target). **(E**–**I)** The expression of METTL14, ZCCHC4, NOP58, IGF2BP2, and IGF2BP23 in PDAC tissues vs the normal (GEPIA2). **(J)** qRT-PCR analysis of METTL14, IGF2BP2, and IGF2BP3 in PDAC cells under different local stiffness niches (n = 3, p < 0.05). **(K**–**N)** Representative immunofluorescence staining (scale bar: 10 μm) and quantification showing increased METTL14 (red) and IGF2BP3 (violet) intensity of MIA-PACA2 cells in high local stiffness groups (n = 3, p < 0.05, vs low local stiffness group). **(O**–**P)** YAP1 mRNA stability assay of MIA-PACA2 cells in high local stiffness niches treated with Actinomycin D (10 μg/mL) after IGF2BP3 perturbation. **(Q**–**R)** Western blot results showing METTL14/IGF2BP3 knockdown or overexpression reciprocally regulate YAP1 protein levels of MIA-PACA2 cells in high local stiffness niches. **(S)** In MIA-PACA2 cells, the cooperative regulation of YAP1 by METTL14 and IGF2BP3 in high local stiff niches, β-actin served as control. Data: mean ± SD, ∗p < 0.05, ∗∗p < 0.01, ∗∗∗p < 0.001, ∗∗∗∗p < 0.0001, and n.s.: denotes not significant. (For interpretation of the references to color in this figure legend, the reader is referred to the Web version of this article.)

**Fig. 8:**
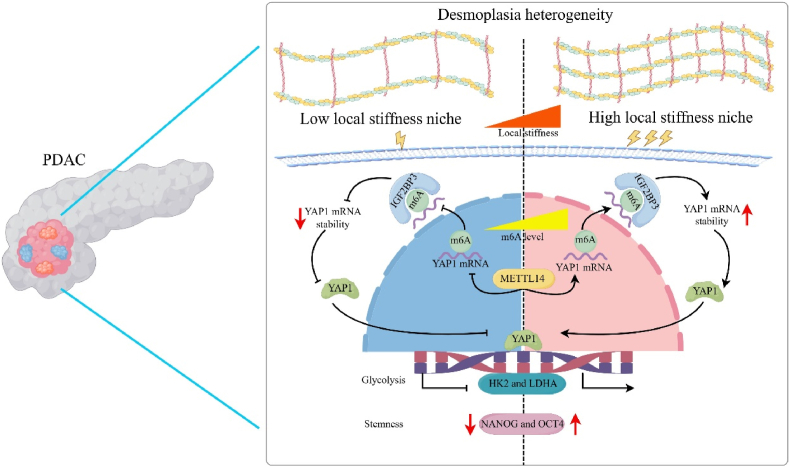
**Graphical illustration of the mechanism events involved in local high stiffness-regulated PDAC stemness.** The PDAC TME is desmoplastic and mechanically heterogeneous, containing low and high local stiffness niches. Persistent stimuli in stiff local niches enhance the level of YAP1 via METTL14/IGF2BP3-suppressed YAP1 mRNA decay, leading to the augmentation of glycolysis and the acquisition of a stem-like phenotype for PDAC cells.

CSCs could maintain relative plasticity by up- or downregulating genes under epigenetic control based on the environment in which they exist [52]. GO analysis of CSCs within PDAC primary and liver metastasis lesions exhibited the regulation of mRNA stability biological process (Fig. 1D and I). The GO analysis based on the DEGs of mechanical sensitivity related to YAP1 (Fig. 6B) also shows enriched BP of methylation of mRNA and regulation of the mRNA stability. The increased YAP1 transcriptional and protein levels have been evaluated by qRT-PCR and immunofluorescence, respectively. This indicates that stiffness heterogeneity may boost the YAP1 level via aberrant m6A modification. Hence, we first predicted the m6A modification sites of YAP1 mRNA by SRAMP (Fig. 7A), and the results showed that there were 9 sites with very high confidence and 8 sites with high confidence, accounting for 58.62 % of the total (Fig. 7B). And the global m6A level in 3D printing biomimetic local niche with low, medium, and high stiffness was further evaluated by dot blotting (Fig. 7C) and immunofluorescence (S-Fig. 7A–B). The quantitative analysis indicates the global m6A level increased with the local stiffness (n = 3, p < 0.05). The m6A modification is determined by “WERs”, including the methyltransferases (writers, W), demethylases (erasers, E), and RNA-binding proteins (readers, R). To further explore the stiffness-induced YAP1 related- “WERs”, we overlap LOX positively correlated genes (R > 0.145, p < 0.05, TCGA-PAAD) in PDAC, YAP1 positively correlated genes (R > 0.145, p < 0.05, TCGA-PAAD) in PDAC, and the predicted WERs of YAP1 mRNA by m6A RM2Target (Fig. 7D). Three “writers” (METTL14, ZCCHC4, and NOP58) and two “readers” (IGF2BP2, and IGF2BP3) were obtained. METTL14, IGF2BP2, and IGF2BP3 are significantly upregulated in the PDAC tissues than the normal (Fig. 7E, H, and I, p < 0.05), while ZCCHC4 and NOP58 are not significant (Fig. 7F, G, p > 0.05). The level of METTL14 and IGF2BP3 detected by qRT-PCR is remarkably elevated for both MIA-PACA2 and CFPAC-1 cells in the high local stiffness niches (Fig. 7J, n = 3, p < 0.05). Therefore, METTL14 and IGF2BP3 were selected. The expression of METTL14 and IGF2BP3 was positively correlated with YAP1 in PDAC (S-Fig. 7C–D, R = 0.80 and 0.74, p < 0.05). The immunofluorescence (Fig. 7K and M, S-Fig. 7Eand G) was performed, and the quantitative analysis aligns with the qRT-PCR results (Fig. 7L and N, S-Fig. 7Fand H, n = 3, p < 0.05). Then, shRNA targeting METTL14 and IGF2BP3 are validated (S-Fig. 7I–J, n = 3, p < 0.05). And RNA immunoprecipitation (RIP) assays of PDAC cells within the high local stiffness group were further carried out. The enrichment of YAP1 mRNA in complexes precipitated is not significantly different with METTL14 antibodies (S-Fig. 7K and M, n = 3, p > 0.05), while it was remarkably elevated with IGF2BP3 antibodies (S-Fig. 7L and N, n = 3, p < 0.05), compared with the IgG. Several investigations have been reported that the m6A level of YAP1 is regulated by METTL3 [33,[53], [54], [55]], while METTL14 functions as a methyltransferase (writer) via interacting with METTL3 [56,57], which may account for METTL14 cannot combine with the YAP1 mRNA. And IGF2BP3, RNA binding proteins (readers), bind to m6A-containing mRNAs and increase the stability. To assess whether the increased level of YAP1 mRNA in the high local stiffness group stems from IGF2BP3-mediated RNA stability, we measured the mRNA lifetime of YAP1 by inhibiting transcription with actinomycin D (Act D) for 0, 2, 4, and 6 h. The half-life (T_1/2_) of YAP1 mRNA is decreased in the IGF2BP3 knocked-down (sh) group compared to the control group (Fig. 7O and S-Fig. 7O). For MIA-PACA2 cells, the sh-IGF2BP3-1 T_1/2_ = 4.77 h, sh-IGF2BP3-2 T_1/2_ = 4.56 h, and sh-NC T_1/2_ = 6.07 h. And for CFPAC-1 cells, the sh-IGF2BP3-1 T_1/2_ = 4.42 h, sh-IGF2BP3-2 T_1/2_ = 4.90 h, and sh-NC T_1/2_ = 5.83 h. While the half-life is extended in the overexpressed (OE) IGF2BP3 group (Fig. 7P and S-Fig. 7P), the OE-IGF2BP3 T_1/2_ = 8.98 h and OE -NC T_1/2_ = 5.25 h for MIA-PACA2 cells, the OE-IGF2BP3 T_1/2_ = 8.41 h and OE -NC T_1/2_ = 4.82 h for CFPAC-1 cells. We further evaluated the protein levels via western blot (Fig. 7Q–R, S-Fig. 8A–B). And the quantitative analysis (S-Fig. 8D–L) reveals that the levels of YAP1 decreased significantly in the sh-METTL14 or sh-IGF2BP3 group (n = 3, p < 0.05), while remarkably increased in OE-METTL14 or OE-IGF2BP3 group (n = 3, p < 0.05). In addition, the level of YAP1 in METTL14/IGF2BP3 co-overexpression groups exceeds the OE-METTL14&sh-IGF2BP3 and sh-METTL14& OE-IGF2BP3 group. While for METTL14/IGF2BP3 co-knock down groups, the level of YAP1 was remarkably decreased (Fig. 7S, S-Fig. 8C, and S-Fig. 8M–N, n = 3, p < 0.05). These demonstrated that the local stiff niche promotes YAP1 expression via METTL14/IGF2BP3-suppressed mRNA decay.

Collectively, we propose a mechano-m6A regulatory axis wherein heterogeneous high local stiffness drives METTL14/IGF2BP3-mediated m6A modification, stabilizing YAP1 transcripts to potentiate glycolysis and subsequent stem-like phenotype augmentation in PDAC. This paradigm may shift “normalizing ECM” treatment strategies towards more precise PDAC local stiffness by targeting mechanical epigenetic vulnerability (Fig. 8).

## 4 Discussion

Cancer stem cells (CSCs) play a primary role in conferring malignancy in pancreatic ductal adenocarcinoma (PDAC) and are increasingly viewed as a functional state influenced by plasticity within the tumor microenvironment (TME) [8]. Our study reveals mechanical heterogeneity, especially local stiff niches, as a critical yet underappreciated organizer of CSCs in PDAC. The mechanics and glycolysis are activated in PDAC CSCs according to the bioinformatics of spatial transcriptomic and single-cell analyses. The Masson’ s Trichrome and Alcian blue staining displayed the uneven desmoplasia degree in PDAC, which reflects the heterogeneous stiffness further detected by the nano-indenter. 3D biomimetic niches matching the low, medium, and high local stiffness in PDAC are constructed by digitallight-processing (DLP) 3D printing and desmoplastic bioink. The PDAC cell stem-like phenotype is boosted by glycolysis via YAP1 which was upregulated in the high local stiffness niches through METTL14/IGF2BP3-suppressed YAP1 mRNA decay. Then, three pivotal advances were listed: (1) PDAC tissues exhibited remarkable stiffness heterogeneity, and the DLP 3D-printing niches resolving stiffness gradients provide higher resolution for studying tumor mechanobiology, which combine with the desmoplastic bioink permits modulation of mechanical properties by adjusting exposure parameter without altering the biochemical ligand density; (2) the local stiff niche is an underestimated amplifier for the tumor stem-like phenotype via metabolic reprogramming in PDAC; (3) YAP1 regulation via m6A (METTL14/IGF2BP3) in stiff niche reveals mechano-epigenetic CSCs plasticity in PDAC mechanical heterogeneous TME. This finding supports the notion that tumor heterogeneity results in treatment inefficiency, as the heterogeneity of 10.13039/501100010577TME leads to cellular heterogeneity through the interactions between tumor cells and the microenvironment.

In recent decades, rapidly accumulating evidence has demonstrated the remarkable remodeling of extracellular matrix (ECM) in TME, including the enrichment, deposition, and cross-linking, resulting in the general stiffening of various tumors [11]. Meanwhile, numerous previous studies have shown that high stiffness promotes malignant behavior, including migration and invasion [58], chemoresistance [59], and stemness [60]. There is a common assumption that the tumor becomes equally stiff throughout. In our previous study, human PDAC tissues were evaluated using a compressive tester, with the compressive modulus found to be approximately 40 kPa [22]. This condition enhances the stemness and drug-resistance of PDAC cells through increased autophagy [12,61]. The process procedure starts from a macro perspective and assumes its texture to be uniform and consistent, while just considering individual variations and ignoring intratumor heterogeneity. Nonetheless, considerable intra-tumoral heterogeneity, a characteristic of cancer, might cause notable differences in the stiffness of local niches within the TME [62]. Wei et al. proposed that the local matrix stiffness in hepatocellular carcinoma varies based on collagen content, which ranges from approximately 1000 to 9000 Pa [63]. And breast cancer displayed a stiffness spectrum varying from around 0.2 to 45 kPa in Tang et al.'s research [42]. Meanwhile, A.J. Rice et al. proposed that stiffening during ECM remodeling occurs at a local scale in the pancreatic intraepithelial neoplasia (PanIN) and the PDAC [64], which further supports the heterogeneity of stiffness within PDAC. Our research identified the heterogeneity of desmoplasia through Masson’ s Trichrome and Alcian blue staining, indicating the heterogeneous local stiffness within the PDAC tumor microenvironment. And the spectrum of stiffness in PDAC spans 200-6 000Pa according to the nano-indenter.

Simultaneously, the tumor stiffness could be measured from multiple viewpoints, such as the cellular scale, tissue scale, and noninvasive visualization methods (e.g., radiological method). And the discrepancies in PDAC stiffness could be attributed to the individual variations, tissue anisotropy, species differences, and diverse evaluation techniques. PDAC tissue from KPC mice measured by atomic force microscope (AFM) exhibited the upper quartile local Young’ s modulus is approximately 4000 Pa, which is an intermediate stiffness in the transition that enhances Epithelial-Mesenchymal Transition (EMT) and drug resistance [64]. Based on the AFM, Liu et al. reported that the stiffness of patient-derived xenograft (PDX) PDAC tissues ranges from 1.7 to 11.5 kPa, and mouse-derived allografts (MDAs) range from 6 to 13 kPa, and the high stiffness (10 kPa) promotes stem-like type and therapy resistance [65]. This is further indicative of the intrinsic heterogeneity among species. The EMT and drug resistance are closely correlated to the acquisition of stem-like phenotypes. Although the stiffness variate, all of them indirectly support our experimental results that mechanical priming in stiff niches endows the acquisition of stem-like phenotype. In addition, noninvasive visualization methods, such as optical coherence tomography (OCT) [66] and acoustic radiation force impulse imaging (ARFI) [67], are employed to measure tumor stiffness. And the Young’ s modulus of human PDAC is about 44.8 ± 5 kPa via harmonic motion elastography (HME), which seems not to exhibit the intratumor heterogeneity [68]. Wherein the stiffness heterogeneity based on ultrasound shear-wave elastography (SWE) has been reported in breast cancer [42]. Integrating multiple PDAC intro-tumor stiffness heterogeneity detection methods with molecular markers to create more accurate early diagnostic tools or construct a “patient-specific” mechanical microenvironment chip to guide personalized treatment decisions is a crucial direction for future research.

The lack of complexity in 2D cultures, the missing of 3D structural organization, and the allogenic TME models in vivo have reduced their relevance to human physiopathology, leading to increased false positive rates [69]. The specific spatial organization of tissue mechanics generates different micro-mechanical environments [70]. And the heterogeneity of TME necessitates models capable of capturing its traits for deeper exploration. The rise of 3D cultures, such as 3D printing, 3D scaffolds, and microfluidics, as a solution to this issue, revolutionized and transformed cancer research. 3D bioprinting is a developing technique with great potential to accurately control the spatial organization of different components, such as biomaterials and bioactive molecules [71]. Micro-extrusion is the main technology employed in 3D printing, especially for cancer, due to its capability to integrate millimeter-scale tissues and multi-material printing, which is difficult to achieve with other bio-fabrication methods [44]. Meanwhile, DLP-based bioprinting is a fast bio-fabrication approach that works with different light-sensitive biomaterials, achieving micron-scale resolution. This technology permits precise orthogonal control over biophysical properties and biochemical cues, making it an ideal method to fabricate a biomimetic model for investigating the biophysical effects on PDAC development. The continuous nature of DLP 3D printing enables the fabrication of scaffolds without structural artificial interfaces, thereby enhancing the structural and mechanical integrity of the printed structures. Additionally, it also allows the digital modulation of mechanical properties in the printed structures by adjusting exposure parameter, which permit the continuous light-based production of complex scaffolds with arbitrary regions [72]. Meanwhile, investigating the biophysical aspects of TME requires 3D matrices that can faithfully mimic native ECM and account for the mechanical heterogeneity owing to both ECM composition and mechanical properties function as key regulators for tumor cell behavior.

PDAC is featured by the enrichment, deposition, and cross-linking of the ECM, a process known as desmoplasia, which leads to increased stiffness. The desmoplastic ECM primarily consists of collagen, especially collagen I (COL I), and glycosaminoglycans (GAGs), particularly hyaluronic acid (HA) [24]. Gelatin methacrylate (GelMA), which comes from denatured COL I and retains the arginine-glycine-aspartate (RGD) motif, boosts cellular functions with remarkable biocompatibility. And hyaluronic acid methacrylate (HAMA) has been utilized in numerous studies as a bioactive material that retains polysaccharide structure. In our previous study, the GelMA&HAMA undergo a reaction to create a stable hybrid hydrogel with methacryloyl bond upon exposure to ultraviolet light (UV), which mimics not only the main component but also the crosslink state of the ECM [22]. Menekse Ermis et al. also developed the GelMA&HAMA-based PDAC model with different stiffness by adjusting the curing time [73]. Hence, the combination was selected for the desmoplastic bioink. And combining the DLP 3D printing, the system permits independent modulation of stiffness through UV cross-linking duration at a constant concentration, which avoids altering the biochemical ligand density. We printed the cylindrical model with four internal holes to promotes material exchange [74]. This design further improves the delivery of materials (including nutrients and oxygen) and facilitates the effective removal of metabolic waste for cells located in both the edge and core regions of the model. This strategy reduces the impact of uneven gradients of nutrients and oxygen when exploring the mechanical cues affecting tumor cells. Meanwhile, the porosity remains unchanged with increasing crosslinking time, which further eliminates the influence of material mass transport. These enable drug testing in pathophysiological contexts, such as a stiffness-responsive nanomedicine delivery system, thereby increasing the translational potential for mechanotype-guided therapy strategies.

Importantly, integrating local niches with different stiffness into a single structure-integrated model indeed makes more sense and is biologically relevant due to the ability to faithfully recapitulate in vivo TME. Several researchers have focused on this strategy. By forming a layered polyacrylamide (PA) structure with a stiff bottom layer and a compliant top layer to mimic heterogeneity of stiffness in TME, which was exploited to investigate the directional migration of cancer cells [75]. A microfluidic device based on the controllable stiffness gradient, which consists of fibronectin-conjugated PA hydrogel with a longitudinal stiffness gradient ranging from about 1 kPa to 40 kPa, is integrated within the cell culture chamber to study the behaviors of glioma cells [76]. However, modulating the local ECM microstructures precisely around specific cells to observe cell-ECM communications in a bulk hydrogel system is challenging [77]. It is difficult to isolate regions with specific stiffness and cells within corresponding units in a single structure-integrated model that has a varying stiffness gradient. This difficulty hinders the detection of differentially expressed genes and proteins—such as those identified through RNA sequencing, qRT-PCR, and western blotting—as well as certain distinct metabolites induced by mechanical cues, which are essential for exploring the underlying mechanisms involved. Meanwhile, C. Liu et al. also pointed out that one gel with a different stiffness ingredient may induce cell migration and other crosstalk between cells in different locations, and the strategy of separating gels of different stiffnesses allows for focusing on the local microenvironment and dissecting the effects of pure local stiffness from a stiffness gradient [78]. The PA-based separate 2D tunable mechanical substrate was exploited to investigate the heterogeneous stiffness of local niches' effect on metastatic organotropism in breast cancer [42]. And similar strategy was also applied in hepatocellular carcinoma to demonstrate that heterogeneous matrix stiffness regulates the cancer stem-like cell phenotype [63]. Hence, the separate strategy combined with the DLP 3D-printing and specific desmoplastic bioink in our research allows for a focus on the local stiffness more purely, thus dissecting the effects and potential mechanisms induced by stiffness heterogeneity in PDAC. We believe our research will provide important basic knowledge for future studies on heterogeneous TME.

The notion that “normalizing ECM” has been proposed as a potential strategy in the treatment of tumors with increased stiffness [14]. Drugs that target the stiff ECM have been effective in investigations and are progressively being introduced into clinical applications [79]. Nonetheless, breaking down the dense ECM might eliminate obstacles to cancer spread [80]. Meanwhile, the soft niches have been clarified to promote brain metastasis in breast cancer [42]. PDAC cells also exhibited a stronger proliferative ability within the local soft niches in our previous research. These lead to a contradiction: given the heterogeneous stiffness within PDAC, chemotherapy combined with treatments targeting stiff matrix might boost the proliferation and metastasis of tumor cells, thereby diminishing the effectiveness of the therapy. Consequently, reducing matrix stiffness indiscriminately with drugs might not be the most optimal treatment targeting stiffness in PDAC. Apart from preventing ECM from stiffening, curbing how cells react to mechanical stimuli offers a more targeted way to counteract the negative impacts of microenvironment stiffening.

YAP1 is an emerging node that connects mechanical cues, metabolism, and tumor cell stem-like phenotype within TME. It is essential for the initiation and progression of several tumors. In breast cancer, tumor cells maintain a stem-like phenotype by activating YAP1 signaling [81], and YAP1 also acts as a mechanical sensor in hepatocellular carcinoma [29]. Meanwhile, the mechanical cues transmitted by signaling pathways also regulate metabolic processes. YAP1 is found to be involved in metabolism regulation, including glycolysis, lipogenesis, and glutaminolysis [82], which metabolic flux exerts an integral role in promoting and maintaining stem-like phenotype in tumors. These are consistent with our results that the local stiff niche regulates the stem-like phenotypes via YAP1-mediated glycolysis and imply that YAP1 could be a therapeutic target. Small-molecule inhibitors that directly target YAP1 have been employed as mechanobiological interventions [83] and have demonstrated therapeutic effects in curbing ovarian cancer advancement [84]. However, mice with HCC tumors did not show a significant reduction in tumor size when given a YAP1 inhibitor [63]. This contradiction may stem from neglecting the upstream factors of mechanical stimulation-mediated YAP1.

Meanwhile, many studies have shown that m6A modifications regulate YAP1 levels. It is reported that m6A-mediated YAP1 boosted migration in gastric cancer [33] and tumorigenesis in chordoma [34]. The decreased m6A impedes YTHDF2-mediated degradation of YAP1 to enhance the tumor cell stemness in breast cancer [85]. Notably, the abnormal m6A modifications were found in varying stiffness to regulate pituitary adenomas [86] and modulate macrophage inflammatory responses [87]. These implications of m6A in mechanical signal decoding. In our research, the regulatory function of mechanic-m6A on YAP1 was identified in response to the PDAC heterogeneous stiffness, in which local stiff niches enhance YAP1 levels to boost the tumor cell stem-like type via METTL14/IGF2BP3-suppressed YAP1 mRNA decay. The mechano-m6A-YAP1 route may explain how TME heterogeneity contributes to cellular heterogeneity through genomic variations to some extent. The epi-transcriptional mechanism may mechanistically explain how mechanical cues create lasting oncogenic states and also expand our understanding of how m6A modifications contribute to PDAC. This insight further supports the development of more targeted therapy strategies that focus on the mechanical transduction pathways involved in tumor cell responses to stiff niches, potentially reducing the adverse effects associated with stiff ECM degradation. It highlights the intricate and dynamic characteristics of the TME, indicating that future studies should thoroughly integrate all elements and crosstalk to reveal deeper molecular mechanisms and create more effective targeted therapies. These also prompt us to investigate the role of other epigenetics, such as DNA methylation, histone modification, and chromatin remodeling, in response to heterogeneous TME mechanics in the future.

Our findings indicate that the mechanical heterogeneity of local niches within PDAC lesions promotes glycolysis via mechano-transduction, which facilitates the acquisition of a stem-like phenotype for tumor cells. Yet, this effort remains challenging due to the limited methods or tools available to reliably isolate and trace cells from specific stiff niches in vivo. The primary PDAC lesions used to test stiffness heterogeneity are early-stage, while advanced lesions may expand the stiffness spectrum. And there is a lack of mechanical testing for PDAC liver metastasis lesions. The current model controls stiffness but not desmoplasia-induced hypoxia and interstitial fluid pressure (IFP), which may co-regulate glycolysis-induced stem-like phenotype. Due to the dynamic nature of ECM degradation and production, the system fails to track the stiffness fluctuations caused by ECM alterations during culture. The structure-integrated units with heterogeneous stiffness and other stromal cells, such as cancer-associated fibroblasts (CAFs) and macrophages, will be incorporated in our next-gen models. And in vivo efficacy of mechano-epigenetic targeting requires testing in genetically engineered PDAC models.

## 5 Conclusion

The research focused on the mechanical heterogeneity within PDAC, which is simulated by local niches with low, medium, and high stiffness based on 3D printing and desmoplastic bioink. Mechanical priming in stiff niches promotes the acquisition of stem-like phenotype by YAP1-induced glycolytic reprogramming. Mechanistically, the expression of YAP1 is mediated by the METTL14/IGF2BP3 suppressed mRNA decay in response to the local stiff niches. This study enhances our understanding of the epigenetic modifications that link tumor biomechanical heterogeneity to the malignant behaviors induced by metabolic reprogramming within the PDAC. These insights could lead to the development of new therapeutic strategies targeting tumor mechanics heterogeneity.

## CRediT authorship contribution statement

**Di Wu:** Writing – review & editing, Writing – original draft, Visualization, Methodology, Funding acquisition, Formal analysis, Data curation, Conceptualization. **Xiaoqi Guan:** Visualization, Funding acquisition, Formal analysis, Data curation. **Tao Yang:** Visualization, Investigation, Formal analysis, Data curation. **Jiashuai Yan:** Formal analysis. **Biwen Zhu:** Formal analysis. **Junchao Zhou:** Formal analysis. **Yibing Guo:** Supervision, Resources, Project administration, Formal analysis. **Yuhua Lu:** Writing – review & editing, Visualization, Supervision, Project administration, Methodology, Funding acquisition.

## Declaration of competing interest

The authors declare that they have no known competing financial interests or personal relationships that could have appeared to influence the work reported in this paper.

## Data Availability

Data will be made available on request.

## Abbreviations

Pancreatic ductal adenocarcinoma: (PDAC)
Cancer stem cells: (CSCs)
Tumor microenvironment: (TME)
Extracellular matrix: (ECM)
Gelatin methacrylate: (GelMA)
Hyaluronic acid methacrylate: (HAMA)
Digitallight-processing: (DLP)
Ultraviolet light: (UV)
Adenosine triphosphate: (ATP)
Nicotinamide adenine dinucleotide phosphate: (NADPH)
Immunohistochemistry: (IHC)
Complementary DNA: (cDNA)
Immunofluorescence: (IF)
Yes-associated protein 1: (YAP1)
N6-methyladenosine: (m6A)
Knockdown: (KD)
Overexpression: (OE)
Actinomycin D: (Act D)
Gemcitabine: (GEM)
2-Deoxy-D-glucose: (2-DG)
Half maximal inhibitory concentration: (IC_50_)
Oligomycin: (Oligo)
Verteporfin: (VP)
Differentially expressed genes: (DEGs)
Gene ontology: (GO)
Kyoto Encyclopedia of Genes and Genomes: (KEGG)
Biological process: (BP)
Glycosaminoglycans: (GAGs)
Hematoxylin-Eosin: (H&E)
Lysyl-oxidase: (LOX)
Overall survival: (OS)
Disease specific survival: (DSS)
Progress free interval: (PFI)
Collagen I: (COL I)
Hyaluronic acid: (HA)
Arginine-glycine-aspartate: (RGD)
Polyacrylamide: (PA)
Epithelial-Mesenchymal Transition: (EMT)
Atomic force microscope: (AFM)
Patient-derived xenograft: (PDX)
Mouse-derived allografts: (MDAs)
Optical coherence tomography: (OCT)
Acoustic radiation force impulse imaging: (ARFI)
Harmonic motion elastography: (HME)
Shear-wave elastography: (SWE)
Interstitial fluid pressure: (IFP)
Cancer-associated fibroblasts: (CAFs)
Pancreatic intraepithelial neoplasia: (PanIN)
