# Supplementary material for: 3D printing-biomimetic local stiff niche enhances glycolysis to boost PDAC cell stem-like phenotype via N6-methyladenosine-suppressed YAP1 mRNA decay
Source: Mater Today Bio. 2025 Aug 5;34:102176. doi: 10.1016/j.mtbio.2025.102176 (PMC12357319; doi:10.1016/j.mtbio.2025.102176)
Supplement: Multimedia component 1 [file mmc1.docx]

**Supplementary figure and legends**

**Supplementary Figure 1. Desmoplasia heterogeneity in PDAC tissues. (A)** Masson′ s trichrome staining highlights collagen deposition (blue) in regions of low (a) and high (b) desmoplastic stroma, sample 2-5. **(B)**Alcian blue staining identifies GAGs (turquoise) with low (a) and high (b) glycosaminoglycan-rich regions, sample 2-5. Scale bar: 200 μm, 50 μm.

**Supplementary Figure 2. Biomechanical and functional validation of desmoplastic-mimetic bioinks**. **(A)** Scanning electron microscopy (SEM) of hydrogels (cell-free) with different stiffness (scale bar: 20 μm). **(B)** Porosity unaffected by stiffness (p > 0.05, n = 3). **(C)** Rheological profiles of cell-laden bioinks: storage modulus (G′) and loss modulus (G″) for PDAC cells-encapsulating hydrogels (including MIA-PACA2 and CFPAC-1 cells). **(D)** Shear-thinning behavior of bioinks containing PDAC cells (shear rate: 0.1-100 s⁻¹).  **(E)** Computer-assisted design (CAD) of the 3D model. **(F)** Young′ s modulus within biomimetic niches containing MIA-PACA2 and CFPAC-1 cells with low (189.7 ± 11.5 Pa and 223.3 ± 29.0 Pa), medium (1982.0 ± 182.1 Pa and 2048.0 ± 3199.9 Pa), and high stiffness (5571.0 ± 466.8 Pa and 5437.0 ± 672.8 Pa) group (n = 5, p < 0.05). **(G)** Swelling test and degradation test **(H)** and quantitative analysis (**I-J**, n = 5, p > 0.05) of the printed tumor niche across local stiffness groups. **(K)** Live/dead assay quantification demonstrates great and comparable viability across local stiffness groups (n = 5, p > 0.05, calcein-AM/PI staining). **(L-M)** H&E staining of 5-day cultured 3D niches showing PDAC cells distribution and matrix architecture (scale bar: 50 μm). Data: mean ± SD, *p < 0.05, **p < 0.01, ***p < 0.001, ****p < 0.0001; n.s.: not significant.

**Supplementary Figure 3. Complementary data: high local stiffness-driven glycolytic reprogramming potentiates PDAC stemness. (A)** Representative immunofluorescence of OCT4 (green) and HK2 (red) in PDAC cells (MIA-PACA2 and CFPAC-1 cells**)** across local stiffness groups (scale bar: 10 μm). **(B)** Quantitative fluorescence intensity analysis of NANOG, OCT4, HK2, and LDHA expression in the low, medium, and high local stiffness groups (n = 3, p < 0.05). **(C)**The dose-response curves and half maximal inhibitory concentration (IC_50_) values of PDAC cells within the low, medium, and high local stiffness groups treated with gemcitabine (GEM, 0.01, 0.1, 1, 10, 100, 1,000 μM) for 48 h (n = 5). **(D)** Representative images of cell spheroid assays for PDAC cells after stimulation of the low, medium, and high local stiffness and quantitative analysis **(E,** n = 3, p < 0.05, scale bar: 50 μm). **(F)** Representative immunohistochemistry images of OCT4 and HK2 in subcutaneously transplanted 3D printed PDAC niches (low local and high local stiffness group, scale bar: 50 μm). **(G)** qRT-PCR analysis of the level of NANOG and OCT4 in rescue experiments of high local stiffness niches group treated with 5 mM 2-Deoxy-D-glucose (2DG) for 12 h or 5μM oligomycin (Oligo) for 12 h (n = 3, p < 0.05, vs high local stiffness + DMSO group). **(H)** The dose-response curves and IC_50_ values of the rescue experiments in response to GEM (n = 5). **(I)** Representative images of cell spheroid assays for the rescue experiments and quantitative analysis (**J**, n = 3, p < 0.05, scale bar: 50 μm). Data: mean ± SD, *p < 0.05, **p < 0.01, ***p < 0.001, ****p < 0.0001; n.s.: not significant.

**Supplementary Figure 4. Clinical relevance of LOX and YAP1 in PDAC prognosis.** **(A-C)** Poor OS/DSS/PFI in high-LOX PDAC patients (p < 0.05, TCGA-PAAD). **(D)** LOX overexpression in PDAC tissues vs normal tissues (p < 0.05, GEPIA2). **(E)** Positive correlation between LOX and YAP1 in PDAC patients (R = 0.87, p < 0.05). **(F)** Higher YAP1 expression in LOX high group vs LOX low group (n = 90 and 89, p < 0.05, TCGA-PAAD). **(G)** YAP1 immunohistochemistry in PDAC tissues and the normal (sample 2-5, scale bar: 50 μm). **(H-J)** YAP1 predicts survival with high AUC (OS = 0.772, DSS = 0.778, PFI = 0.898). Data: mean ± SD, *p < 0.05, **p < 0.01, ***p < 0.001, ****p< 0.0001; n.s.: not significant.

**Supplementary Figure 5. Complementary data: YAP1 is closely linked to tumor stiffness and predicts poor prognosis in PDAC. (A)** qRT-PCR analysis shows elevated YAP1 mRNA levels in PDAC cells (MIA-PACA2 and CFPAC-1 cells) in high local stiffness group vs low local stiffness group (n = 3; p < 0.05). **(B)** Representative immunofluorescence images and heatmap of YAP1 in CFPAC-1 cells across the local stiffness group, scale bar :10 μm. **(C)** Representative immunohistochemistry images of YAP1 in subcutaneous transplanted 3D printed PDAC niches (low local and high local stiffness group, scale bar: 50 μm). Data: mean ± SD, *p < 0.05, **p < 0.01, ***p < 0.001, ****p < 0.0001, and n.s.: denotes not significant.

**Supplementary Figure 6. Complementary data: YAP1 mediates high local stiffness-dependent glycolytic reprogramming in PDAC. (A)** Inhibition of YAP1 with Verteporfin (VP, 1 μg/mL, 12 h) reduces HK2 and LDHA mRNA expression in high local stiffness group, while YAP1 activator PY60 (10 μM, 12 h) reverses this effect (n = 3, p < 0.05, vs high local stiffness + control group). **(B)** Representative Immunofluorescence images of HK2 (red) and LDHA (green) in VP/PY60-treated CFPAC-1 cells in high local stiffness niches (scale bar: 10 μm). **(C)** Quantitative analysis of fluorescence intensity confirms VP suppresses while PY60 rescues HK2/LDHA expression (n = 3, p < 0.05, vs high local stiffness + control group). Data: mean ± SD, *p < 0.05, **p < 0.01, ***p < 0.001, ****p < 0.0001, and n.s.: denotes not significant.

**Supplementary Figure 7. Functional validation of METTL14/IGF2BP3 suppressed the decay of YAP1 mRNA in high local stiffness niches. (A-B)** Immunofluorescence staining (scale bar: 10 μm) and quantification showing increased global mRNA m6A levels in high local stiffness groups (n = 3, p < 0.05, vs low local stiffness group). **(C)** Positive correlation between YAP1 and METTL14 (R = 0.80, p < 0.05, GEPIA2). **(D)** Positive correlation between YAP1 and IGF2BP3 (R = 0.74, p < 0.05, GEPIA2). **(E-H)** Immunofluorescence staining (scale bar: 10 μm) and quantification showing high local stiffness-induced METTL14 and IGF2BP3 upregulation in CFPAC-1 cells (n = 3, p < 0.05, vs low local stiffness group). **(I-J)** shRNA validation targeting METTL14 (sh1-3) and IGF2BP3 (sh1-3) by qRT-PCR (n = 3, p < 0.05, vs sh-NC). **(K-N)** RNA immunoprecipitation (RIP) assay of MIA-PACA2 and CFPAC-1 cells in high local stiffness group with METTL14 (n = 3, p > 0.05) and IGF2BP3 (n = 3, p < 0.05) antibodies. **(O-P)** YAP1 mRNA stability assay of CFPAC-1 cells in high local stiffness niches treated with Actinomycin D (10 μg/mL) after IGF2BP3 perturbation. Data: mean ± SD, *p < 0.05, **p < 0.01, ***p < 0.001, ****p < 0.0001, and n.s.: denotes not significant.

**Supplementary Figure 8. Complementary data: Functional validation of METTL14/IGF2BP3 suppressed the decay of YAP1 mRNA in high local stiffness niches. (A-C)** METTL14/IGF2BP3 modulation regulates YAP1 protein of CFPAC-1 cells in high local stiffness group (Western blot, β-actin served as control). **(D-G)** Quantification showing METTL14 knockdown reduces YAP1, while overexpression elevates YAP1 in MIA-PACA2 and CFPAC-1 cells (n = 3, p < 0.05, vs control group). **(H-L)** Quantitative analysis of IGF2BP3 perturbation modulates YAP1 levels in both cell lines (n = 3, p < 0.05, vs control group)**. (M-N)** Quantification of combined METTL14 and IGF2BP3 knockdown in regulating YAP1 in high local stiffness niches (n = 3, p < 0.05). Data: mean ± SD, *p < 0.05, **p < 0.01, ***p < 0.001, ****p < 0.0001, and n.s.: denotes not significant.

**Supplementary Figure 1**

**
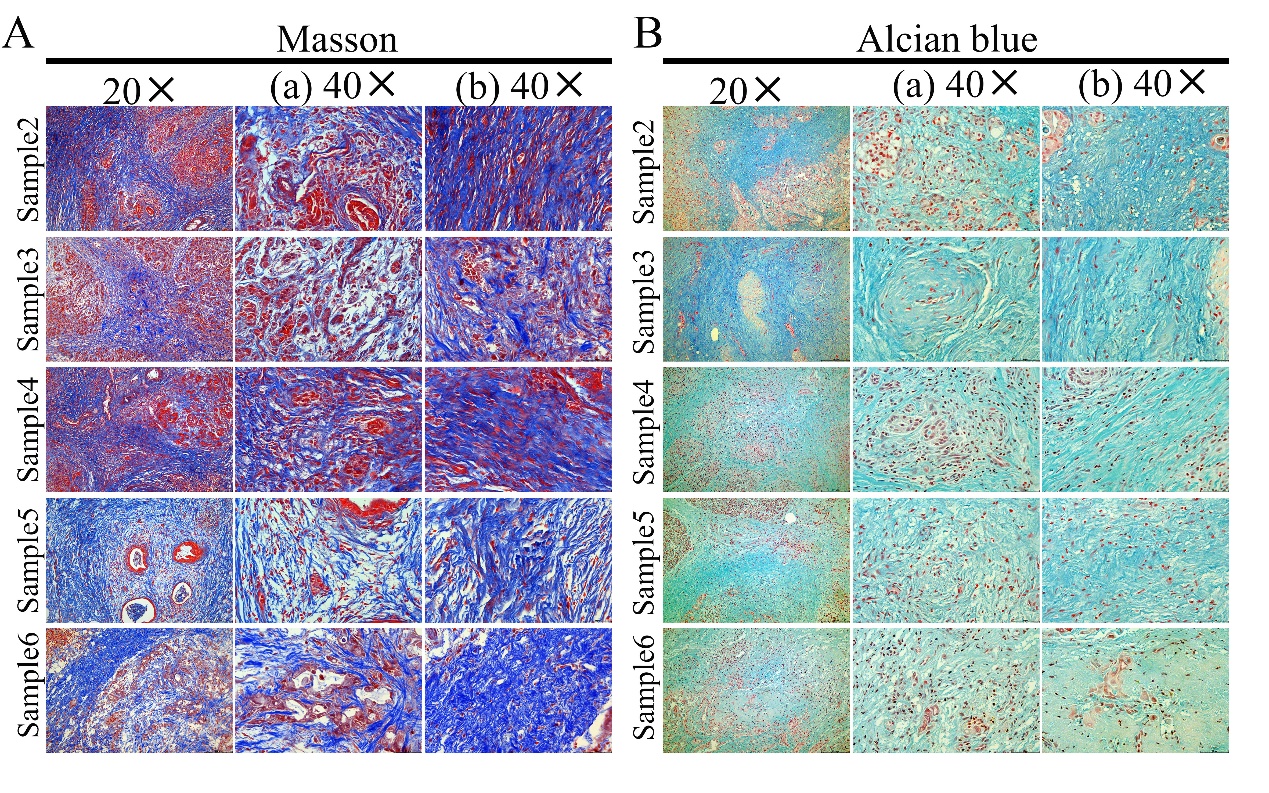
**

**Supplementary Figure 2**

**
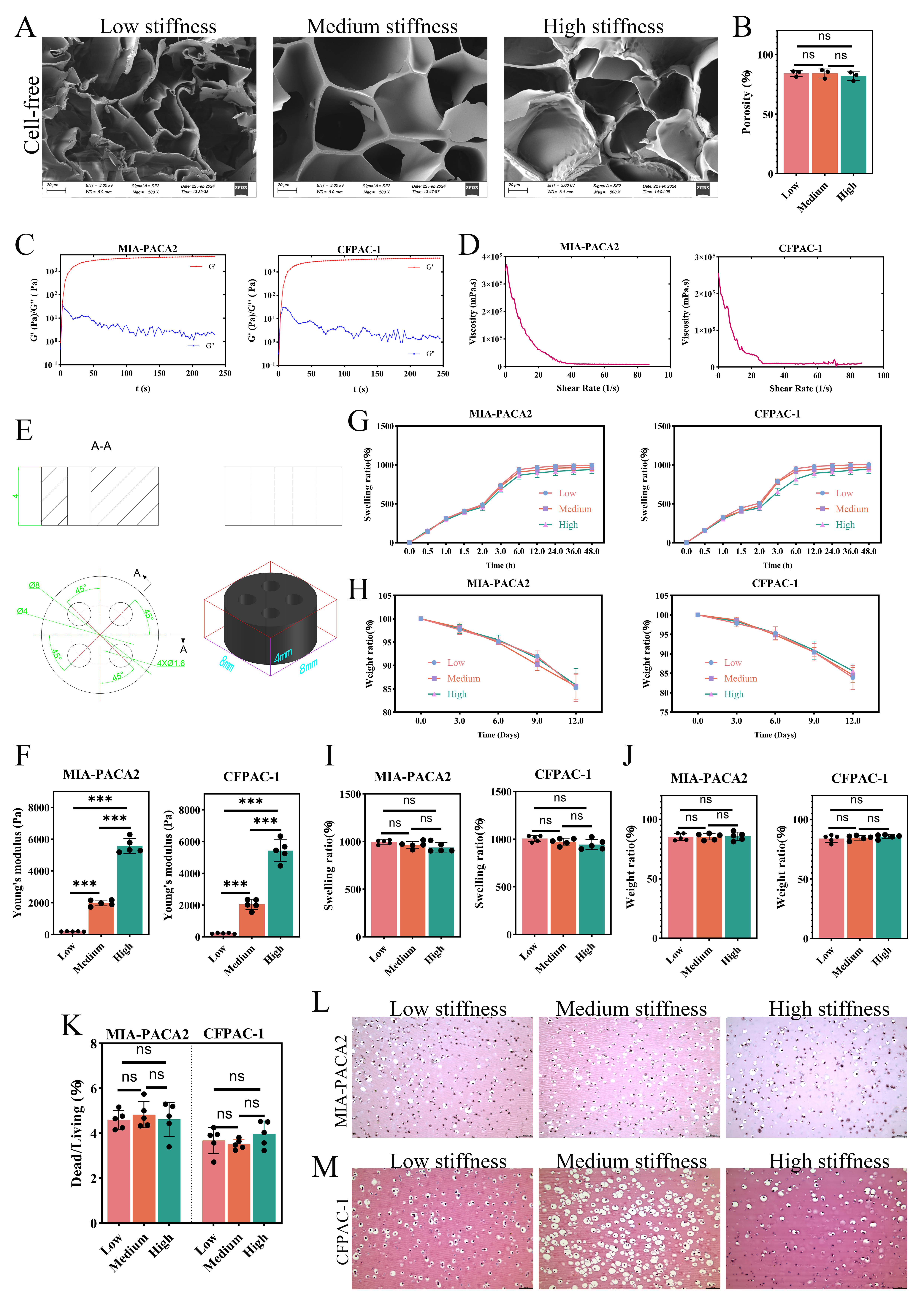
**

**Supplementary Figure 3**

**
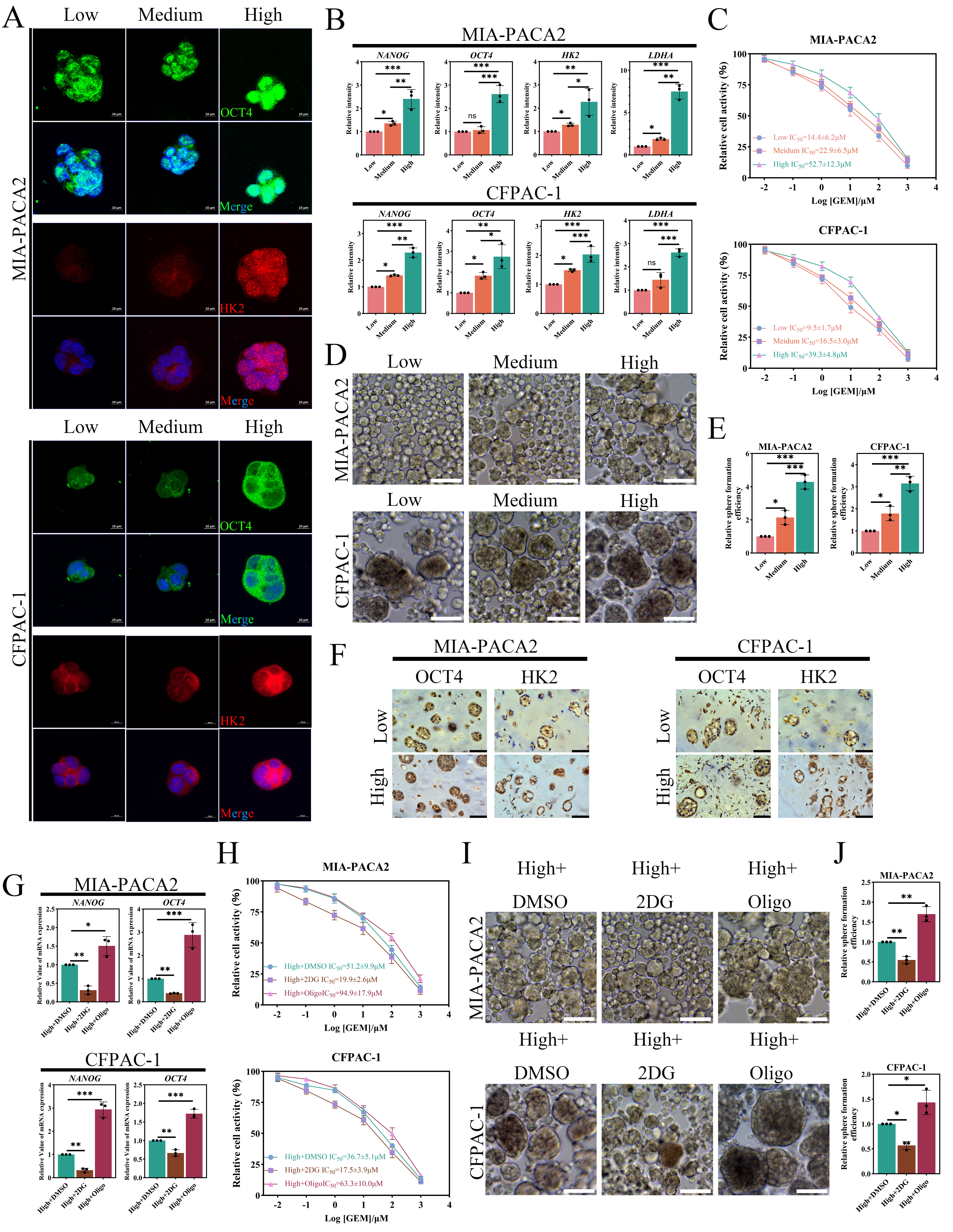
**

**Supplementary Figure 4**

**
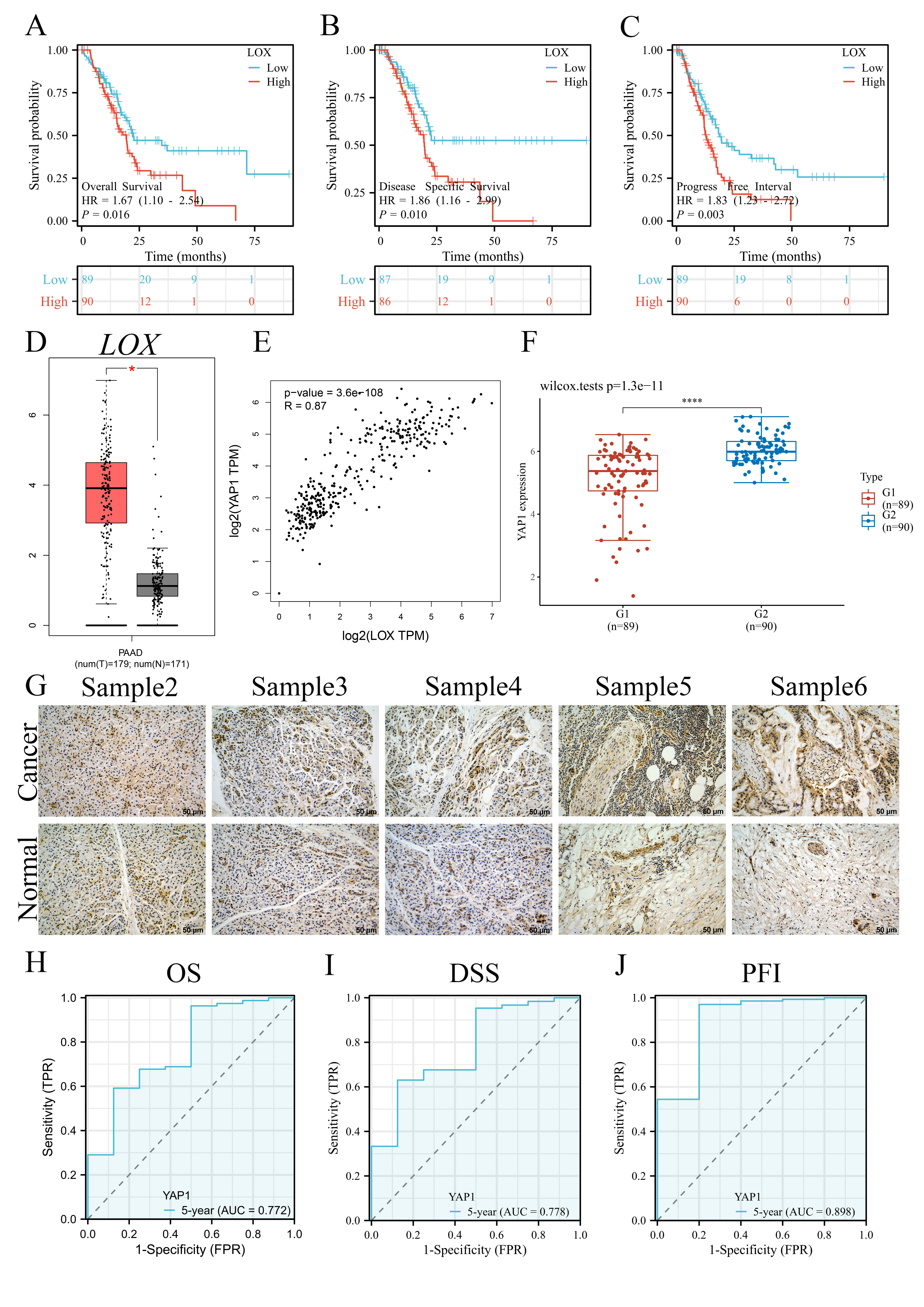
**

**Supplementary Figure 5**

**
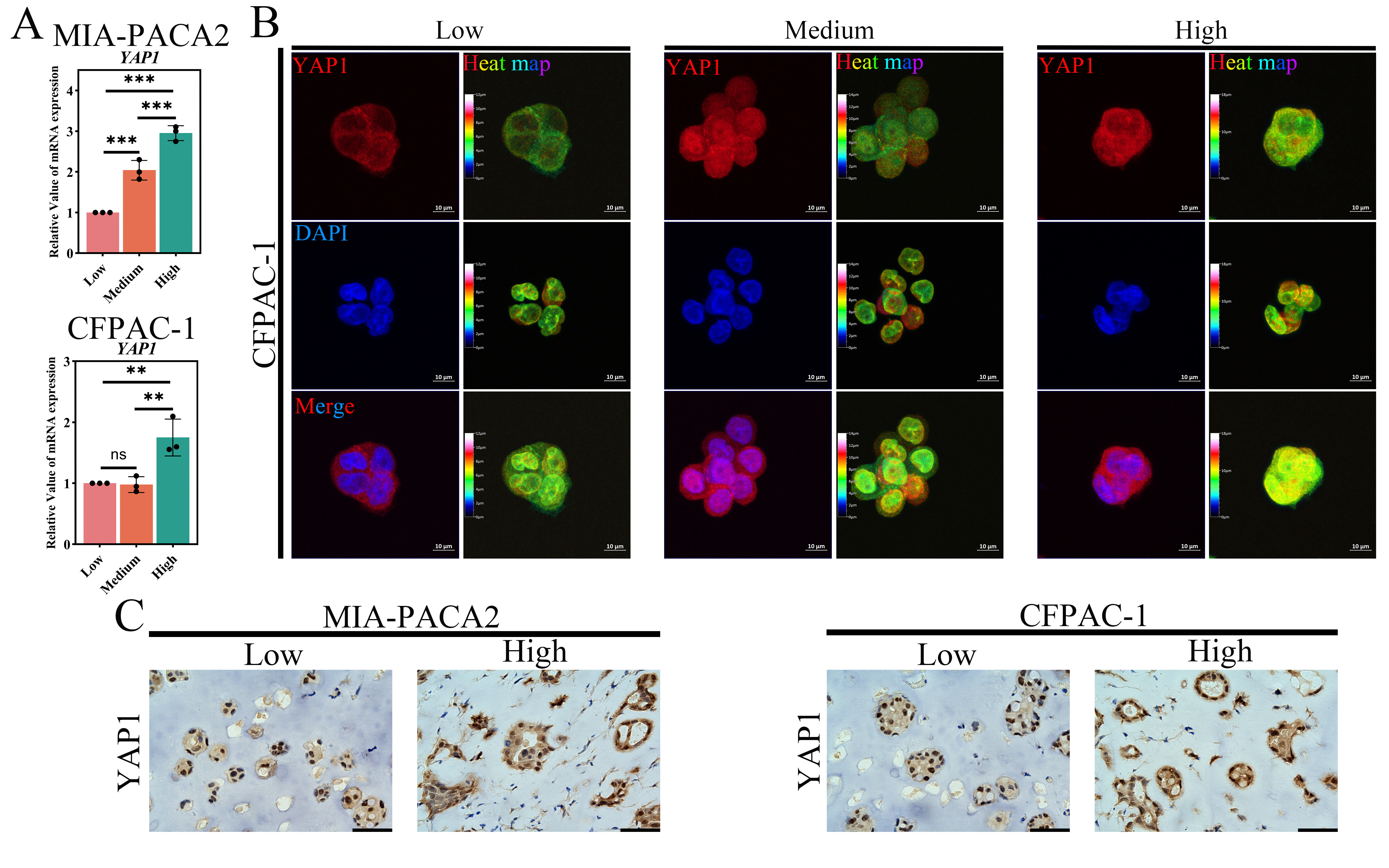
**

**Supplementary Figure 6**

**
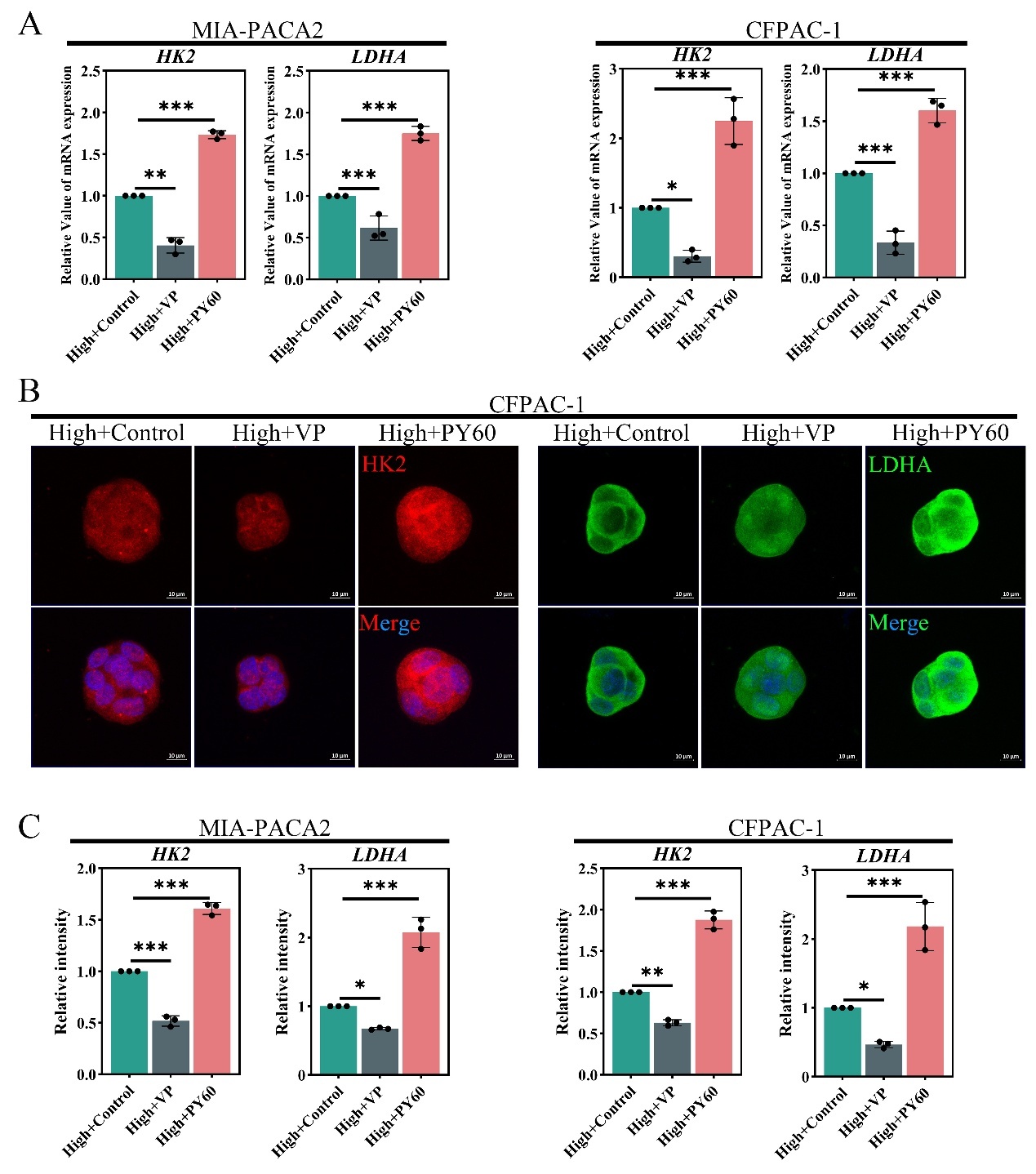
**

**Supplementary Figure 7**

**
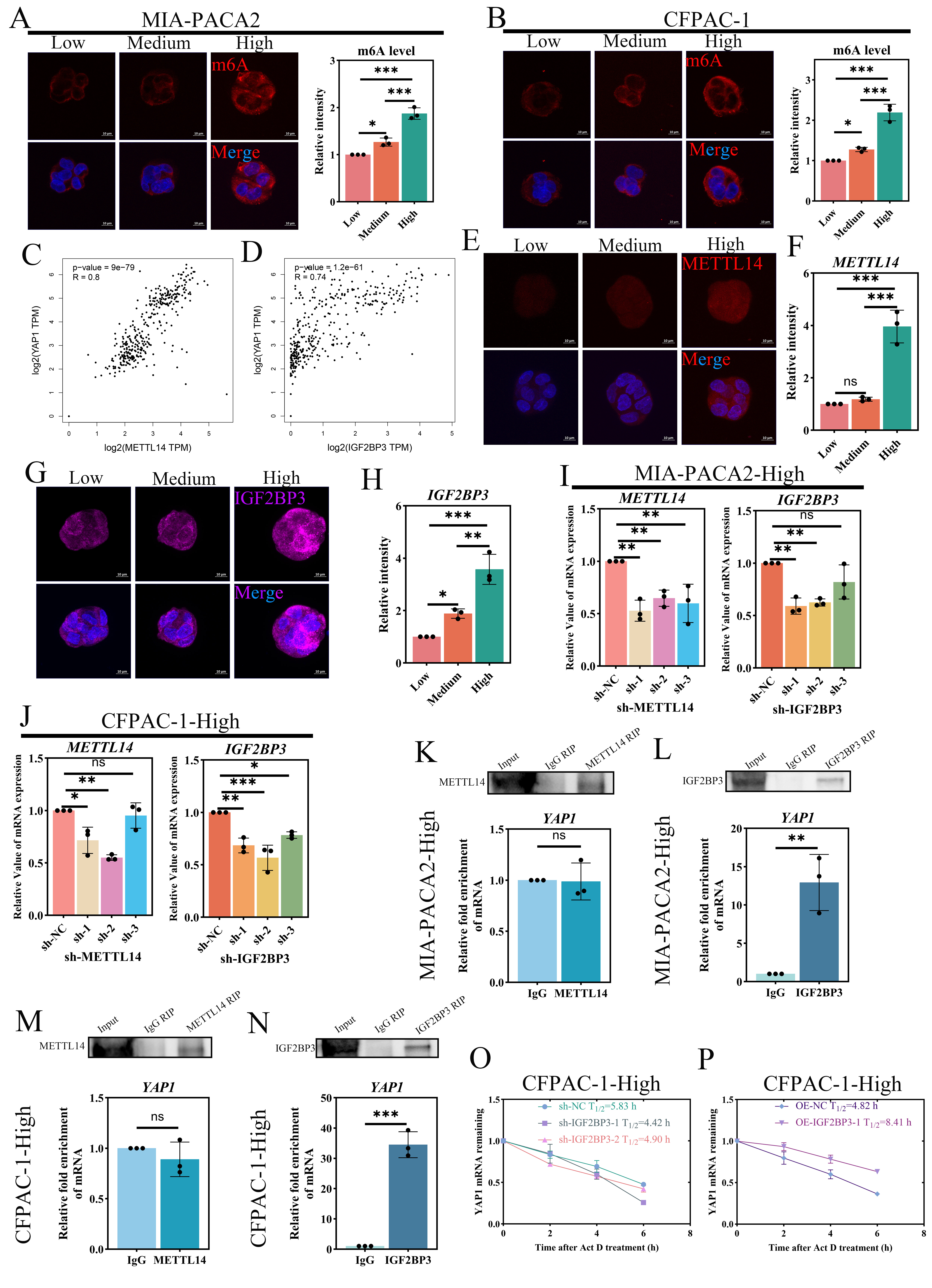
**

**Supplementary Figure 8**

**
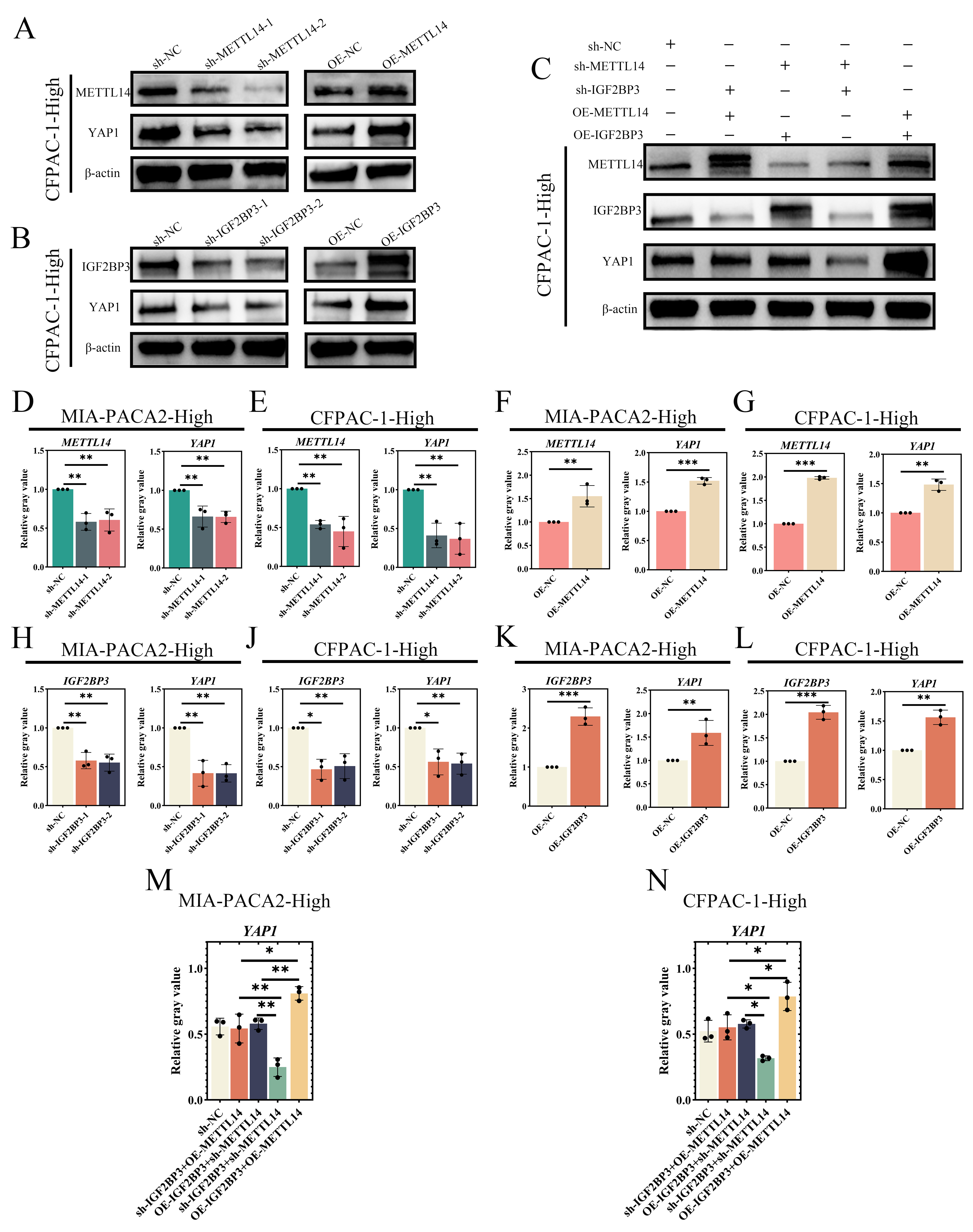
**
